# Mathematical Model of a Telomerase Transcriptional Regulatory Network Developed by Cell-Based Screening: Analysis of Inhibitor Effects and Telomerase Expression Mechanisms

**DOI:** 10.1371/journal.pcbi.1003448

**Published:** 2014-02-13

**Authors:** Alan E. Bilsland, Katrina Stevenson, Yu Liu, Stacey Hoare, Claire J. Cairney, Jon Roffey, W. Nicol Keith

**Affiliations:** 1 Institute of Cancer Sciences, University of Glasgow, Cancer Research UK Beatson Laboratories, Bearsden, Glasgow, United Kingdom; 2 Cancer Research Technology Ltd., Wolfson Institute for Biomedical Research, London, United Kingdom; University of Tokyo, Japan

## Abstract

Cancer cells depend on transcription of telomerase reverse transcriptase (*TERT*). Many transcription factors affect *TERT*, though regulation occurs in context of a broader network. Network effects on telomerase regulation have not been investigated, though deeper understanding of *TERT* transcription requires a systems view. However, control over individual interactions in complex networks is not easily achievable. Mathematical modelling provides an attractive approach for analysis of complex systems and some models may prove useful in systems pharmacology approaches to drug discovery. In this report, we used transfection screening to test interactions among 14 *TERT* regulatory transcription factors and their respective promoters in ovarian cancer cells. The results were used to generate a network model of *TERT* transcription and to implement a dynamic Boolean model whose steady states were analysed. Modelled effects of signal transduction inhibitors successfully predicted *TERT* repression by Src-family inhibitor SU6656 and lack of repression by ERK inhibitor FR180204, results confirmed by RT-QPCR analysis of endogenous *TERT* expression in treated cells. Modelled effects of GSK3 inhibitor 6-bromoindirubin-3′-oxime (BIO) predicted unstable *TERT* repression dependent on noise and expression of *JUN*, corresponding with observations from a previous study. *MYC* expression is critical in *TERT* activation in the model, consistent with its well known function in endogenous *TERT* regulation. Loss of *MYC* caused complete *TERT* suppression in our model, substantially rescued only by co-suppression of *AR*. Interestingly expression was easily rescued under modelled Ets-factor gain of function, as occurs in *TERT* promoter mutation. RNAi targeting *AR*, *JUN*, *MXD1*, *SP3*, or *TP53*, showed that AR suppression does rescue endogenous *TERT* expression following *MYC* knockdown in these cells and *SP3* or *TP53* siRNA also cause partial recovery. The model therefore successfully predicted several aspects of *TERT* regulation including previously unknown mechanisms. An extrapolation suggests that a dominant stimulatory system may programme *TERT* for transcriptional stability.

## Author Summary

Tumour cells acquire the ability to divide and multiply indefinitely whereas normal cells can undergo only a limited number of divisions. The switch to immortalisation of the tumour cell is dependent on maintaining the integrity of telomere DNA which forms chromosome ends and is achieved through activation of the telomerase enzyme by turning on synthesis of the *TERT* gene, which is usually silenced in normal cells. Suppressing telomerase is toxic to cancer cells and it is widely believed that understanding *TERT* regulation could lead to potential cancer therapies. Previous studies have identified many of the factors which individually contribute to activate or repress *TERT* levels in cancer cells. However, transcription factors do not behave in isolation in cells, but rather as a complex co-operative network displaying inter-regulation. Therefore, full understanding of *TERT* regulation will require a broader view of the transcriptional network. In this paper we take a computational modelling approach to study *TERT* regulation at the network level. We tested interactions between 14 *TERT*-regulatory factors in an ovarian cancer cell line using a screening approach and developed a model to analyse which network interventions were able to silence *TERT*.

## Introduction

Immortalisation is a hallmark of cancer commonly achieved by transcriptional reactivation of the telomerase reverse transcriptase gene *TERT* [1]. Multiple transcription factors modulate *TERT* and previous studies have identified many of those which individually contribute to activate or repress telomerase levels in cancer cells, resulting in a highly complex picture of *TERT* regulation [2]. In cancer cells lacking tight control of chromatin mediated silencing present in normal cells, a few factors such as c-Myc and Sp1 may act as “master regulators”. However, many other factors bind the *TERT* promoter, co-operating with these and other pathways, and acting together to ensure telomerase expression in a wide variety of cancer cells.

It is increasingly recognised that transcription factors do not behave in isolation, but rather as a complex co-operative network [3] and *TERT* expression most likely also occurs in this context [4], [5]. For example, *TERT* transcriptional suppression by different *TP53* family members is mediated through distinct combinations of binding sites for c-Myc, Sp1 and E2F-family proteins [6], while E2F family members themselves activate or suppress *TERT* in a cell-specific manner [7]. Furthermore, WT1 dependent *TERT* repression in renal cancer cells involves upregulated expression of *TERT* repressors *SMAD3* and *JUN*, as well as down-regulation of activators *AP-2* and *NFX1* [8].

We previously observed that GSK3 inhibition causes widespread *TERT* promoter remodelling and that GSK3 inhibited ovarian cancer cells show long-term unstable telomerase suppression, correlating with altered protein expression and oscillation of several *TERT* regulatory factors, particularly c-Jun [4]. Thus, upstream telomerase regulatory interventions are mediated through multiple effects at the promoter but can also cause broader network effects. In addition, *TERT* regulators such as p53 and NF-κB are also known to exhibit complex dynamic behaviour such as oscillating expression under certain conditions [4], [9].

These dynamic effects may be of relevance for therapeutic interventions directed at telomerase expression including gene therapy and pathway therapeutics. For example, it is likely that many different combinations of active signalling pathways and transcription factors are compatible with *TERT* expression. Therefore, *TERT* expressed under different “network states” may be more or less susceptible to targeting by specific agents. Hence, there is a need for systems-level understanding of telomerase control.

Approaches such as network inference or enrichment analysis are useful in identification of functional relations in omics data [5], [10]–[13]. However, in-silico mathematical models of pathway dynamics are also proving increasingly useful to understand organising principles of signal transduction [14]. In one example, integration of proteomics data with sensitivity analysis of a kinetic model of ERK pathway activation suggested that PC12 cell differentiation relies on distributed control [15]. Modelling may also prove useful in translational systems pharmacology as, for example, in probing signalling mechanisms which give rise to resistance to anti-HER2 antibodies [16] or identification of NGF pathway targets [17].

Here, we report the first mathematical model of *TERT* regulation. We developed a classical Boolean threshold network model involving *TERT* and 14 of its regulatory transcription factors. Boolean networks (BN) are among the simplest dynamic modelling tools but are useful models of transcriptional networks [18], [19]. The general BN modelling framework is discussed in detail in the materials and methods section. Briefly, BN offer a low resolution modelling solution comprising a set of nodes (genes) connected in a network, each of which takes one of two states (on or off). In each run-time step, active nodes positively or negatively regulate the on/off state of other nodes as determined by a rule table. Node states are updated on each step. In this study, we use the rule that if fewer repressors than activators of any node are on in any time step, then that node will become or remain active on the next step. Alternatively, if repressors dominate, the node will be turned off. As discussed in materials and methods, classical BN models always converge either to steady states or oscillations. Characterisation of these is a principal method of model analysis.

Though their dynamics are simple, BN have been used to investigate a range of cellular pathways [20]–[22]. Advantages include ease of modelling constitutive activation or suppression of nodes by modifying their rule tables or of investigating particular interactions by adding or deleting them from the model. BN are well suited for first models of complex systems such as the current model of *TERT* where few kinetic parameters are known.

We adopted a transfection screening approach in A2780 ovarian cancer cells for development of our core model interactions. We obtained promoter reporters and expression vectors for 14 transcription factor regulators of *TERT* and transfected these against each other, testing all pair-wise interactions. The updating rule was then applied to the defined interactions and model steady states evaluated. The model successfully predicted *TERT* transcriptional responses to several signalling inhibitors and reproduced the well documented role of *MYC* expression as a master regulator of *TERT*. Thus, cell based screening may be a useful general approach for production of BN.

Further analysis of the role of *MYC* led to the finding that *AR* co-suppression is able to reverse *MYC* dependent *TERT* suppression in A2780 cells. We also tested the addition of Ets-factor gain of function at *TERT* as has been reported to occur in *TERT* promoter mutations [23]–[25]. Under these conditions, *TERT* suppression by *MYC* inhibition is fragile, suggesting a role for Ets-factors in promoting *TERT* expression robustness. An extrapolation from topological analysis of the model suggests that *TERT* may be hard wired for transcriptional stability in cancer cells which has possible implications for pathway therapeutics approaches targeting telomerase.

## Results

### Development of *TERT* network model by transfection screening

In order to develop a BN model, it was first necessary to define a static structural model of the *TERT* transcriptional network. As described in supplemental file Text S1, we initially tested several literature-derived networks before deciding to employ the novel approach of reporter screening to define an interactions network for *TERT* transcriptional regulation at the level of a single cell line. The literature-derived models had, in general, poor performance in reporting dynamic behaviour relevant to *TERT* expression which may be because the interactions are curated from experiments performed in divergent contexts using a range of different cell lines and reagents. We assembled a panel of luciferase reporter vectors comprising 1 kb proximal human gene promoter regions for a set of 14 previously reported *TERT* regulatory transcription factors. Correspondingly, we obtained a panel of expression vectors for the same factors. These comprised *SP1*, *MYCN*, *RELA*, *MYC*, *HIF1A*, *FOS*, *STAT3*, *AR*, *JUN*, *TP53*, *E2F1*, *MXD1*, *SP3*, and *NR2F2* (table 1). The sources of promoter and expression constructs are given in materials and methods. Our *TERT* reporter construct has previously been reported [26]. We interrogated all pair-wise interactions by cotransfecting each expression vector against the entire promoter panel.

**Table 1:** Accession numbers of all human genes from the model.

Gene	Gene ID
*AR*	367
*JUN*	3725
*TP53*	7157
*SP1*	6667
*E2F1*	1869
*MYCN*	4613
*MXD1*	4084
*RELA*	5970
*MYC*	4609
*FOS*	2353
*HIF1A*	3091
*SP3*	6670
*STAT3*	6774
*NR2F2*	7026
*TERT*	7015

**Figure 1:**
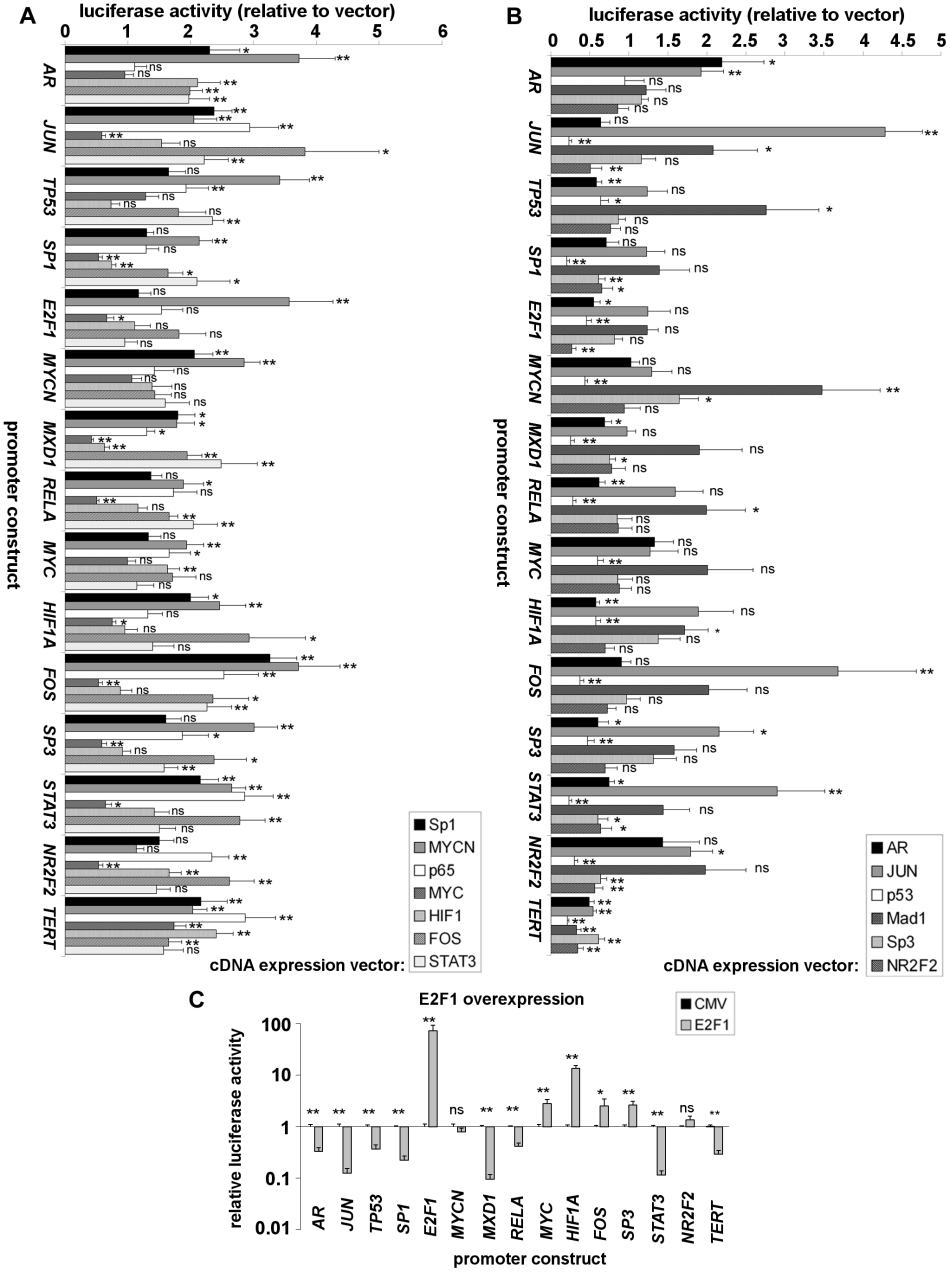
Definition of the *TERT* transcriptional neighbourhood in A2780 cells by transfection screening. (A), overexpression of *TERT* activators. A2780 cells were transfected with the luciferase reporters shown on the vertical axis. Each reporter was co-transfected alongside vector control or transcription factor expression plasmid shown in the right hand boxes. Each bar type represents a different expression vector relative to control. 48 h post-transfection, promoter activities were analysed by luciferase assay. (B), overexpression of *TERT* repressors, transfected as in (A). (C), overexpression of *E2F1* against the promoter panel, transfected as above. Because of the very strong self-regulatory effect on its own promoter, *E2F1* is shown on a different scale and separately from the other *TERT* repressors. Mean ± SEM of 3 experiments (ns: not significant; *: p<0.5; **: p<0.01).

Each interaction identified from the transfection data was incorporated in the network model as an activating interaction mediated by a given transcription factor if the overexpressed transcription factor increased the activity of a promoter. If promoter activity was decreased, the interaction was defined as repressive. These results are shown in figure 1. Note that the effect of overexpressing *E2F1* is shown separately from the other *TERT* repressors on a log scale in figure 1C because of its very strong self-regulatory effect (73.5-fold activation). Effects at the *TERT* promoter (right hand side of each panel) were in the expected direction for all factors except *FOS*. The reason for this discrepancy is not clear, but may reflect the different construct and cell line [27]. We initially tested several candidate network models assembled by using different criteria for inclusion of an interaction in the network and implementing the BN rules on each network. The threshold rule governing model dynamics is given in materials and methods. Briefly, the rule is activation-dominant: for any node, unless more of its repressors than activators are on at any time step, the node will be active on the next time step.

We tested a range of cutoff values based on fold-change in promoter activity and significance (ANOVA) for inclusion of individual interactions from the transfection screen and implemented the rules on each resulting network. The results of this model-fitting exercise are also detailed in supplemental file Text S1. The best results were obtained when we selected cutoffs of minimum 1.5-fold change in reporter activity with p<0.01 (in figure 1, interactions meeting the cutoff are marked **).

A single relaxation of these cutoffs was made for the effect of *STAT3* at the *TERT* promoter. Although regulation tended in the expected direction, it was not significant. Other studies have shown that *STAT3* does activate *TERT* expression [28] and we have previously detected *STAT3* binding to the *TERT* promoter by ChIP analysis in A2780 cells [4] and we re-performed this analysis here (supplemental file Text S1). We retained *STAT3* as a *TERT* activator since our model performs better with its inclusion. We discuss this decision and the role of *STAT3* in our model in detail in supplemental file Text S1, along with results obtained using a network in which *STAT3* is excluded. As detailed in the file, the *STAT3*-deleted model still captured most behaviour we describe in this report, though the model was more aligned with some experiments when it was included. The selected model comprises 92 total interactions of which 50 are activating and 42 are repressive.

We performed literature searching with inclusion/exclusion criteria detailed in materials and methods for each of these individual interactions. We found 47 previously reported interactions (excluding *STAT3* activation of *TERT* since this was retained according to a modelling decision). The cumulative hypergeometric probability of this overlap was p(≥47) = 1.24×10^−4^. 35 interactions were found to be in agreement with our results (table 2) [27], [29]–[63]. 12 previously reported interactions were non-concordant with our results, having different effect directions (table 3) [27], [49], [55], [64]–[72]. The reasons for these differences may be because of the use of different cell lines or constructs. However, the cumulative binomial probability of this concordance is p(≥35) = 5.44×10^−4^. Hence, the overlap between our screen and the literature was highly significant. The remaining 44 interactions we identified are given in table 4. We apologise if we have overlooked the work of any authors who have previously demonstrated these interactions according to our literature search criteria. If we are made aware of any such study, we will endeavour to cite it in any future publications relating to our model. We next analysed the model dynamic behaviour in more detail.

**Table 2:** Common, concordant interactions in literature and data-derived models.

From→To	Literature Effect	Reference
*AR→TP53*	Inhibition	Rokhlin et al (2005) [53]
*AR→TERT*	Inhibition	Moehren et al (2008) [48]
*JUN→JUN*	Activation	Angel et al (1988) [31]
*JUN→TERT*	Inhibition	Takakura et al (2005) [27]
*TP53→MYC*	Inhibition	Ho et al (2005) [39]
*TP53→E2F1*	Inhibition	Ookawa et al (2001) [52]
*TP53→SP1*	Inhibition	Tapias et al (2008) [56]
*TP53→FOS*	Inhibition	Kley et al (1992) [45]
*TP53→TERT*	Inhibition	Kanaya et al (2000) [44]
*SP1→JUN*	Activation	Chen et al (1994) [33]
*SP1→MYCN*	Activation	Hossain et al (2012) [40]
*SP1→FOS*	Activation	Duan et al (1998) [36]
*SP1→TERT*	Activation	Kyo et al (2000) [46]
*E2F1→E2F1*	Activation	Johnson et al (1994) [43]
*E2F1→HIF1A*	Activation	Sengupta et al (2011) [54]
*E2F1→AR*	Inhibition	Davis et al (2006) [35]
*E2F1→MYC*	Activation	Thalmeier et al (1989) [57]
*E2F1→TERT*	Inhibition	Crowe et al (2001) [34]
*MYCN→TP53*	Activation	Chen et al (2010) [32]
*MYCN→E2F1*	Activation	Oliver et al (2003) [51]
*MYCN→TERT*	Activation	Mac et al (2000) [47]
*MXD1→TERT*	Inhibition	Oh et al (2000) [50]
*RELA→JUN*	Activation	Xing et al (2013) [61]
*RELA→TP53*	Activation	Hsu and Lee (2011) [41]
*RELA→FOS*	Activation	Anest et al (2004) [30]
*RELA→TERT*	Activation	Gizard et al (2011) [38]
*MYC→JUN*	Inhibition	Zeller et al (2006) [63]
*MYC→TERT*	Activation	Wu et al (1999) [60]
*HIF1A→TERT*	Activation	Anderson et al (2006) [29]
*SP3→SP1*	Inhibition	Nicolás et al (2003) [49]
*SP3→TERT*	Inhibition	Wooten-Blanks et al (2007) [59]
*STAT3→JUN*	Activation	Durant et al (2010) [37]
*STAT3→FOS*	Activation	Yang et al (2003) [62]
*STAT3→MXD1*	Activation	Jiang et al (2008) [42]
*NR2F2→TERT*	Inhibition	Wang et al (2004) [58]

**Table 3:** Common, non-concordant interactions in literature and data-derived models.

From→To	Literature Effect	Reference
*JUN→AR*	Inhibition	Yuan et al (2004) [72]
*JUN→FOS*	Inhibition	Schönthal et al (1989) [70]
*TP53→NR2F2*	Activation	Neilsen et al (2011) [68]
*TP53→STAT3*	Activation	Kim et al (2009) [67]
*E2F1→TP53*	Activation	Choi et al (2002) [66]
*E2F1→SP1*	Activation	Nicolás et al (2003) [49]
*E2F1→SP3*	Inhibition	Tapias et al (2008)b [55]
*MYCN→MYCN*	Inhibition	Sivak et al (1997) [71]
*HIF1A→MXD1*	Activation	Cho et al (2013) [65]
*STAT3→TP53*	Inhibition	Niu et al (2005) [69]
*NR2F2→E2F1*	Activation	Chen et al (2012) [64]
*FOS→TERT*	Inhibition	Takakura et al (2005) [27]

**Table 4:** Candidate novel interactions included from the screen.

From→To	Effect
*AR→RELA*	Inhibition
*AR→HIF1A*	Inhibition
*JUN→STAT3*	Activation
*TP53→JUN*	Inhibition
*TP53→MXD1*	Inhibition
*TP53→RELA*	Inhibition
*TP53→HIF1A*	Inhibition
*TP53→SP3*	Inhibition
*SP1→STAT3*	Activation
*E2F1→MXD1*	Inhibition
*E2F1→RELA*	Inhibition
*E2F1→JUN*	Inhibition
*E2F1→STAT3*	Inhibition
*MYCN→AR*	Activation
*MYCN→JUN*	Activation
*MYCN→SP1*	Activation
*MYCN→MYC*	Activation
*MYCN→HIF1A*	Activation
*MYCN→FOS*	Activation
*MYCN→SP3*	Activation
*MYCN→STAT3*	Activation
*MXD1→MYCN*	Activation
*RELA→NR2F2*	Activation
*MYC→SP1*	Inhibition
*MYC→MXD1*	Inhibition
*MYC→RELA*	Inhibition
*MYC→FOS*	Inhibition
*MYC→SP3*	Inhibition
*MYC→NR2F2*	Inhibition
*HIF1A→MYC*	Activation
*HIF1A→NR2F2*	Activation
*HIF1A→AR*	Activation
*FOS→AR*	Activation
*FOS→MXD1*	Activation
*FOS→RELA*	Activation
*FOS→STAT3*	Activation
*FOS→NR2F2*	Activation
*SP3→NR2F2*	Inhibition
*STAT3→AR*	Activation
*STAT3→RELA*	Activation
*NR2F2→JUN*	Inhibition
*NR2F2→NR2F2*	Inhibition

### Analysis of the basal model dynamics

**Figure 2:**
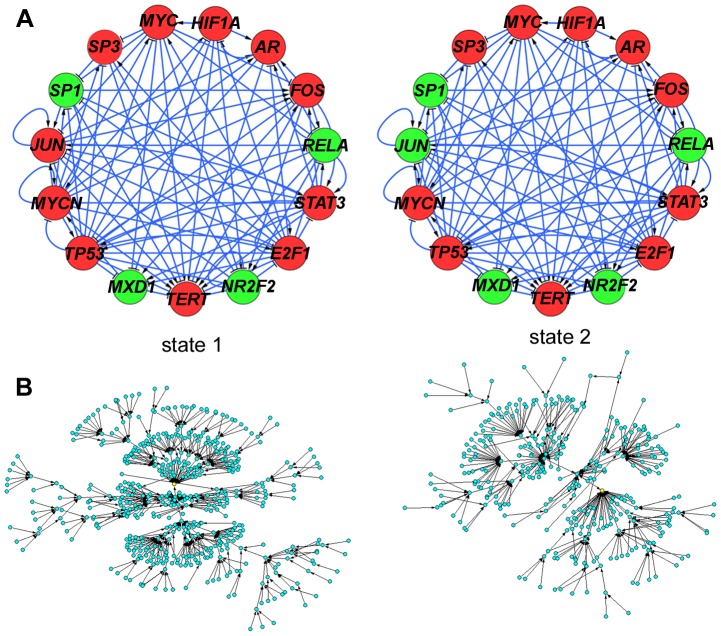
Topology, steady states, and statespace structure of the *TERT* transcriptional neighbourhood model. (A), topology and steady states of the basal *TERT* model. Transfection screening data were used to assign activating or repressive network interactions according to the direction of regulation of each promoter and using the cut-offs of minimum fold-change 1.5 up- or down-regulation of promoter activity and p-value (ANOVA)<0.01. Topology of the final model was visualised in Cytoscape [105]. Arrows indicate activation, T-shape indicates repression. Left and right panels show steady states 1 and 2, respectively. Red colour indicates the node is on, green colour indicates the node is off in each steady state. (B), core statespace structure of the model. Statespace was calculated by brute force and visualised in Pajek [103]. Basins of attraction were extracted as weak components of the statespace. To visualise the core structure, all nodes with in-degree ≥1 were extracted as new networks from each weak component and visualised with transient states in blue and attractor states in yellow. Left panel corresponds to state 1, right panel corresponds to state 2.

The BN model has 32768 total dynamic states, representing the 2^15^ possible combinations of on/off states across all 15 nodes in the model. As noted above, classical BN always converge to either steady states or oscillations. 32766 states are transient states evolving to the 2 steady states of our basal model shown in figure 2A, along with the structure of the network model. *TERT* is active in both steady states, which are highly similar, differing only in *JUN* activation (in the figure, red indicates on, whereas green indicates off). Therefore, telomerase is “stably expressed” in the model, in line with the stable telomerase expression and telomere maintenance that we previously observed in these cells over 6 months in culture [4].

For all model variants in this paper we have performed statespace analysis [73]. This involves sequentially treating each of the 32768 total dynamic states as the current model state, then for each node determining how many of its activators and repressors are currently on. This in turn determines the next state of each node and hence the next overall model state. In this way, every possible state transition is calculated. The approach is only feasible for relatively small networks, but allows identification of all steady states without the need to run multiple simulations starting from different initial conditions.

Since each individual state transits to exactly one next state, the statespace itself can be represented as a network (figure 2B). Here, each node represents a single model state and the arrows represent transitions between them. The single yellow nodes near the centre of each network are the respective steady states shown in figure 2A. All other nodes are transient states evolving toward these. Note that the 32768-state system is too large to show, so a truncated core structure is shown in figure 2B as described in the legend. Steady state 1 (figure 2A, left) dominates, with 20156 associated states which flow to it. If the model is initialised and run from any of these states, it will evolve to steady state 1. 12612 states are associated to steady state 2 (figure 2A, right).

This analysis provides an overview of global dynamics of the model by considering path lengths through statespace. Since no *TERT*-off attractors exist, if perturbed to an “outer state”, the model must return to a *TERT*-on state. Long paths indicate that the model takes longer to reach a steady state. In our model, most system states evolve very rapidly toward the steady states which have *TERT* active. The longest paths are 9 steps and 8 steps for steady states 1 and 2, respectively. Hence, the model exhibits a high degree of global stability with respect to *TERT* activation. Specifically, if the model is transiently perturbed to turn off *TERT*, this will be rapidly reversed. This is an interesting feature of the model, although clearly a qualitative BN model cannot be used to predict the rate of reversal in cells. However, telomere lengths and telomerase expression are stable over greater than 6 months in culture in A2780 cells, suggesting that any noise that does occur must indeed be reversed rapidly enough to facilitate ongoing telomere maintenance [4].

### Modelling inhibitor effects on the *TERT* transcriptional network

**Figure 3:**
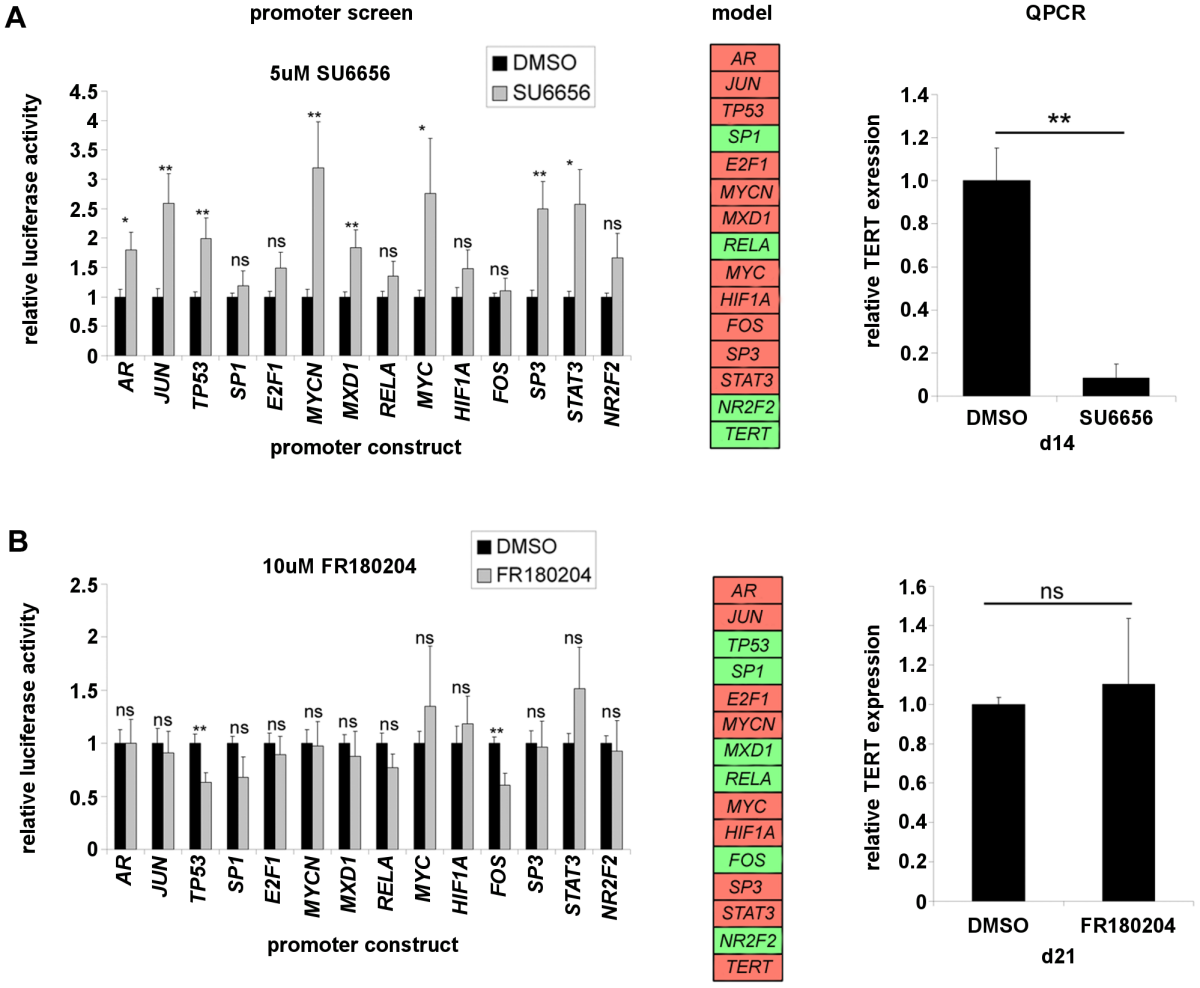
Modelling inhibitor effects on the *TERT* transcriptional neighbourhood. A2780 cells were transfected with each luciferase reporter shown and 32(A), 5 µM SU6656, (B), 10 µM FR180204. Left panels show mean ± SEM of 3 experiments (ns: not significant; *: p<0.5; **: p<0.01). Central panels: luciferase assay results meeting model cut-off of FC>1.5, p<0.01 were modelled as rule table modifications. Heat-map representation of new model steady states obtained by setting rule tables for constitutive activation or suppression at those nodes significantly affected in the luciferase assay. Red colour indicates the node is on, green colour indicates the node is off. Right panels: analysis of *TERT* expression after repeat inhibitor treatments. Control and treated samples from treatment time points shown were analysed by RT-QPCR for *TERT* expression normalised to RPS15. Mean ± SEM of *TERT* expression in treated cells relative to control from three experiments (ns: not significant; **: p<0.01).

It is possible that different states exist in the endogenous *TERT* transcriptional network some of which are more compatible for *TERT* silencing by particular approaches than others. It is of interest to evaluate this question in the modelling context and to compare model behaviour with actual regulation of endogenous *TERT* under signalling interventions. We therefore examined effects of several small molecule kinase inhibitors on the transcription factor promoter panel and incorporated these effects into our model. The compound IUPAC names are given in table 5. A2780 cells were transfected with the promoter panel and 32 h post-transfection were treated for 16 h with either the Src-family inhibitor SU6656 (5 µM), the ERK inhibitor FR180204 (10 µM), or the GSK3 inhibitor 6-bromoindirubin-3′-oxime (BIO, 5 µM). Effects of SU6656 and FR180204 on the promoter panel are shown in the leftmost panels of figures 3A and 3B, respectively.

**Table 5:** IUPAC names and CAS numbers of the compounds used in the study.

Compound	IUPAC name	CAS
SU6656	(3*Z*)-*N*,*N*-Dimethyl-2-oxo-3-(4,5,6,7-tetrahydro-1H-indol-2-ylmethylidene)-2,3-dihydro-1H-indole-5-sulfonamide	330161-87-0
BIO	(3Z)-6-bromo-3-[3-(hydroxyamino)indol-2-ylidene]-1H-indol-2-one	667463-62-9
FR180204	5-(2-Phenyl-pyrazolo[1,5-*a*]pyridin-3-yl)-1*H*-pyrazolo[3,4-*c*]pyridazin-3-ylamine	865362-74-9

SU6656 significantly stimulated several promoter constructs. However, only 5 constructs achieved the cutoffs for inclusion in the model (as before, FC>1.5 and p<0.01). These were *JUN* (FC 2.59; p 0.0048), *TP53* (FC 1.99, p 0.0092), *MYCN* (FC 3.19, p 0.0089), *MXD1* (FC 1.84, p 0.0081) and *SP3* (FC 2.5, p 0.005). Hence, SU6656 had a strong stimulatory effect on the promoters of several *TERT* repressors. In contrast, FR180204 significantly inhibited only two promoters, both of which achieved the cutoffs. These were *TP53* (FC 0.63, p 0.0083) and *FOS* (FC 0.6, p 0.0037).

To model these results for SU6656, the updating rules for *JUN*, *TP53*, *MYCN*, *MXD1* and *SP3* were modified to result in constitutive activity: these nodes were set to the on state independent of any combination of their upstream activators or repressors. Similarly, those of *TP53* and *FOS* were modified for constitutive repression in the case of FR180204. The corresponding steady states resulting from these model changes were then characterised. Both sets of modifications produced a single steady state (shown as heat-maps in the centre panels of figure 3A and 3B).

The modelled effect of SU6656 on the network results in a steady state with *TERT*-off. Hence, the model predicts *TERT* repression by SU6656. In the case of FR180204, a single *TERT* activator and a single repressor are both inhibited. In the new steady state, *TERT* remains on and differs from basal state 1, above, only in *TP53* status. This result could be interpreted either as a prediction of no change or of activation. These alternatives cannot be readily discriminated in this modelling framework.

We next tested the effects of each compound on endogenous *TERT* expression in repeat treatment schedules. A2780 cells were treated twice weekly with SU6656 (5 µM), or FR180204 (10 µM) for 2 weeks (SU6656), or 3 weeks (FR180204). Compounds were not removed between treatments. In the case of FR180204, both control and treated cells were maintained in continuous log-phase for the 3 week treatment duration. However, 5 µM SU6656 induced a complete and sustained growth arrest and the treated cells could be maintained with ongoing treatment for only 2 weeks in this state (not shown).

RT-QPCR analysis revealed profound suppression of *TERT* levels in day 14 SU6656-treated A2780 (right panel, figure 3A). Treated cells had *TERT* levels 8.4% those of control levels. In contrast, in day 21 FR180204 treated cells, no change in *TERT* was observed (figure 3B, right panel). In treated cells *TERT* mRNA levels were 110% of control, but this was not significant. The endogenous effect of both compounds was therefore in line with modelling predictions. To our knowledge this is the first report in which a Boolean network model of a transcriptional network has successfully predicted the outcome of a signalling intervention in respect of gene expression.

**Figure 4:**
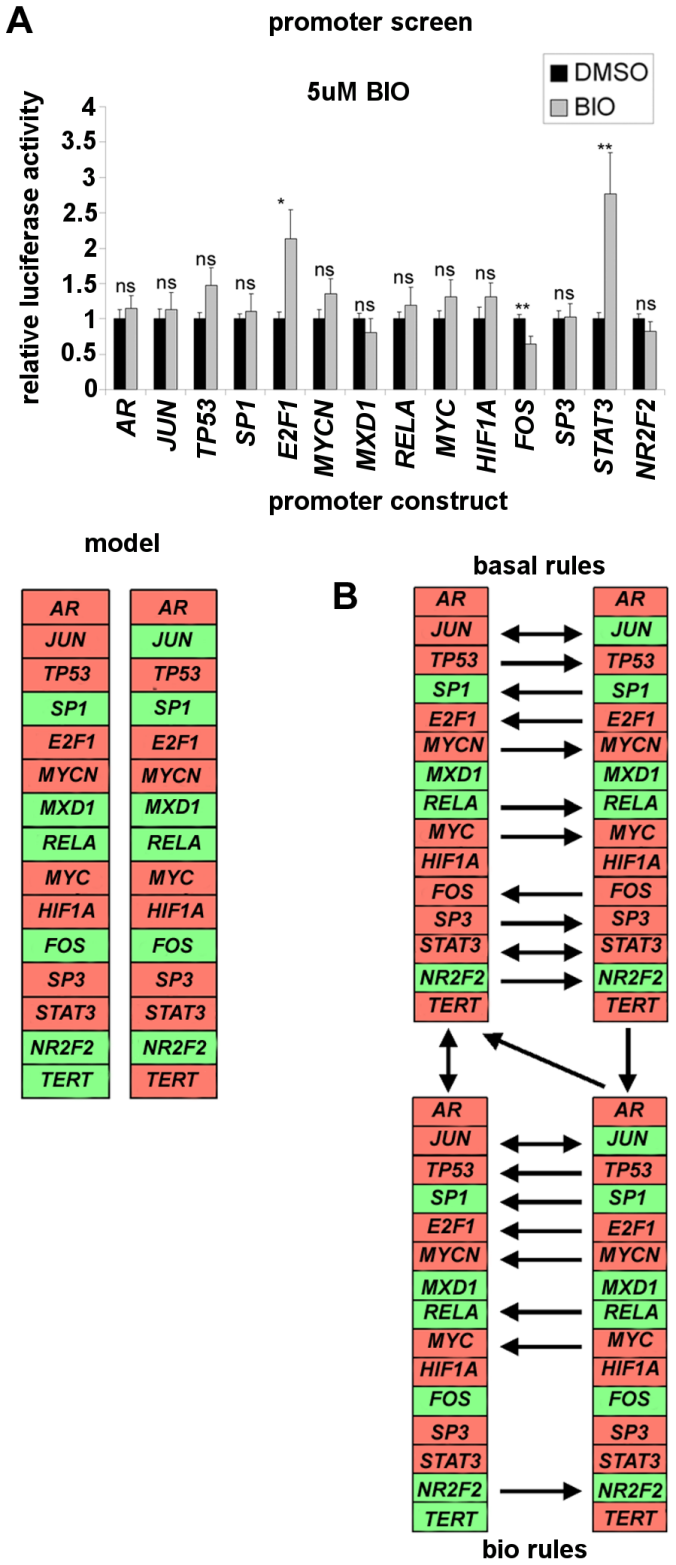
Simulated effects of GSK3 inhibition and network noise on *TERT* transcription. (A), Effect of BIO on the transcription factor promoter panel and simulation in the model. Top panel: A2780 cells were transfected with each luciferase reporter. 32 h later transfectants were treated for 16 h prior to luciferase assay with DMSO or 5 µM BIO. Luciferase assay results meeting model cut-off of FC>1.5, p<0.01 (Fos and *STAT3*) were modelled as rule table modifications. Lower panel: heat-map representation of new steady states obtained by setting Fos to be constitutively suppressed and *STAT3* to be constitutively active. Red colour indicates the node is on, green colour indicates the node is off. (B), noise simulation by the bit-flip method under basal or BIO modified rules in the model. State coherence [73] of each attractor under the model basal rule-set (top) or BIO simulation (bottom) was evaluated as described in the text. Heat-maps of both attractors under either rule-set are shown, with colouration as above. Horizontal arrows between attractor states indicate that a transient state change of the adjacent node caused a shift to the alternate steady state. Vertical and diagonal arrows indicate the state changes resulting from the rule-set change (basal → BIO, or the reverse).

The effect of treating the transfected promoter panel for 16 h with 5 µM GSK3 inhibitor BIO is shown in the top panel of figure 4A. BIO significantly affected the promoters of *E2F1*, *FOS* and *STAT3*. However, only *FOS* (FC 0.64, p 0.0088) and *STAT3* (FC 2.77, p 0.0017) met the model cutoffs. As above, the rule-tables for these nodes were modified to simulate constitutive repression of *FOS* and constitutive activation of *STAT3*.

We have previously reported the effect on *TERT* levels of very long term culture in the presence of BIO [4]. We found that *TERT* is initially suppressed, reaching minimal levels at 3 weeks treatment. Expression subsequently recovers slowly over a period of months, though levels remain low enough to achieve telomere shortening. However, *TERT* expression eventually recovers sufficiently for brief telomere re-elongation, before its levels and telomere length are again suppressed. During this treatment period, levels of *JUN* protein are found to oscillate over a very wide range.

Analysis of the model under BIO modified rules reveals two steady states (figure 4A, heat map labelled “model”). In the dominant BIO state 1 (associated with 26002 system states), *TERT* is off. In BIO state 2 (associated with 6766 system states), *TERT* is on. Therefore, modelling of the effect of GSK3 inhibition on the transcription factor network panel predicts that two conflicting network states are possible under BIO treatment: *TERT* suppressed or not. As with the steady states of the basal model, the key difference between these states is in activity of *JUN*.

### Noise influences *TERT* repression under simulated GSK3 inhibition

To explore the modelling result for BIO in more detail, we examined the impact of simulating transient noise in either the basal model or under the BIO rule modifications. We adopted the “bit-flip” approach for noise simulation: the objective is to model impact of a transient change in each node on the stability of a steady state of interest. The system is initialised with a single state change at one node relative to the steady state of interest (for example, *JUN* on→off relative to steady state 1 of either basal or BIO models). The model is then run to steady state and it is determined whether the transiently modified state returns to the original steady state or not. This is performed sequentially for each individual node and each steady state.

As shown by the arrows representing steady state shifts induced by each node in figure 4B (top), the basal model is readily influenced by simulated noise. State 2 (top right) can be reached from State 1 (top left) through a transient change on 8/14 nodes in the network. However, the reverse transition can be achieved through noise at only 5/14 factors. Therefore, although state 1 associates with the larger fraction of statespace, under noise a switch to steady state 2 is likely. In contrast, the main GSK3 inhibited state 1 (bottom left) is highly resistant to noise with only 2/14 factors able to affect a shift to state 2 (bottom right). Interestingly, the reverse transition to the *TERT*-off state (BIO state 1) is readily achieved through transient changes at 7/14 factors. Importantly, noise at *JUN* is able to promote any shift.

We also examined the effect of a rule-set shift (basal→BIO, or the reverse) in determining which steady states are visited (vertical and diagonal arrows between top and bottom panels). This is intended to model effects of BIO treatment and effect wear-off applied to the different network states. When initialised in basal state 1, application of the BIO rule-set results in evolution to BIO state 1 (*TERT* repressed). However, from basal state 2, the BIO rule modification causes evolution to BIO state 2 (*TERT* active). In the reverse rule shift, both BIO simulated states evolve to basal state 1.

Extrapolating to the biological case, we interpret these results as a model prediction that a significant subset of cells may be at least transiently resistant to *TERT* suppression under BIO treatment (in the model, entering BIO state 2 from basal state 2). However, over time, noise could promote a network state compatible with *TERT* suppression. Therefore, *TERT* levels should initially decrease over time [4]. The impact of *JUN* in mediating the effects of BIO may be central. The model predicts that network noise selects for *JUN* over-expression under BIO treatment (as more cells move to BIO state 1). We did observe progressive up-regulation of *JUN* under long term BIO treatment up to 4 months treatment, after which an oscillation is observed [4]. Up-regulation of *JUN* was also previously found to be important in mediating mesenchymal stem cell differentiation through GSK3α inhibition [74]. However, very high levels of *JUN* may be poorly tolerated. If mechanisms exist to reduce *JUN* expression under such conditions, we suggest that unstable *TERT* expression will result.

### Subsystems in the *TERT* transcriptional network

One key reason to model complex systems is to interrogate potential functional roles of underlying structures in overall system behaviour. In the context of *TERT* it is of interest to attempt to define critical hubs for interventions to stably repress telomerase, and to identify how these cooperate to affect stability. We have previously proposed that stable activation or repression of *TERT* expression using pathway specific inhibitors which affect expression and activity of diverse transcription factors may depend partly on the structure and states of the transcriptional network and, in particular, on the relative effects on feedforward subsystems [4]. We next used our model to further investigate this hypothesis.

**Figure 5:**
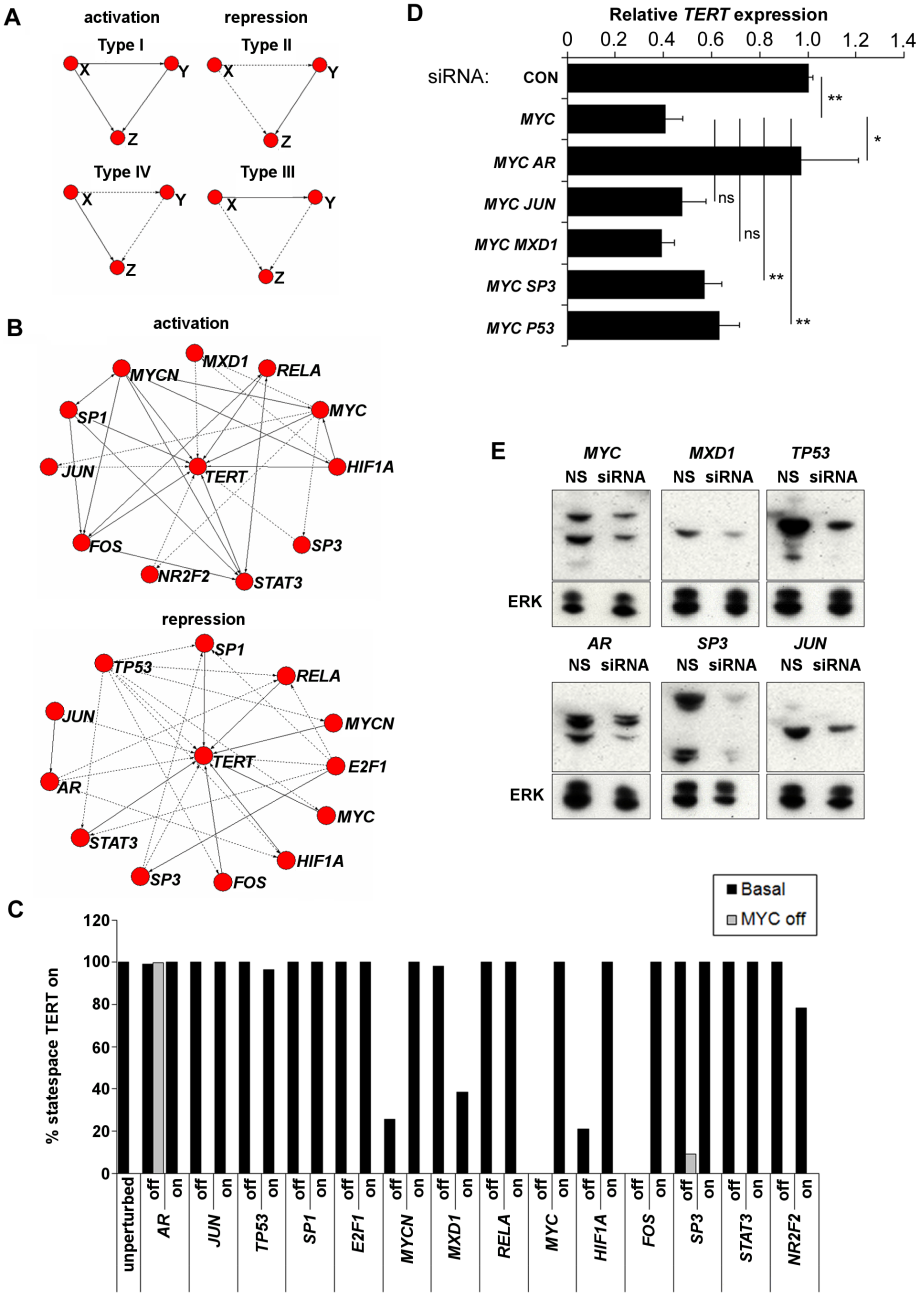
Topological analysis of the *TERT* model, and prediction of robust *MYC* dependent *TERT* repression. (A), structure of FFL types I–IV. Structures visualised in Pajek [103]. Bold lines indicate activation, dashed lines indicate repression. X, Y represent generalised transcription factors, Z represents a regulated gene. (B), activation and repression modules in the *TERT* transcriptional neighbourhood model. Subnetworks were extracted from the main model and visualised in Pajek [103]. Extraction was achieved as described in materials and methods. As an indicator of topological importance, node betweenness centralities were calculated and are given in table 6. Additionally, we calculated flow betweenness which is not dependent only on geodesics [77]. (C), Effect of single- and double-node targeting on *TERT* on-states. Rule-sets for each node were modified in turn individually (black bars) to simulate constitutive repression or activation. For each rule-set change, statespace was derived and the proportion of system states evolving to attractor states with *TERT* stably on was quantified. The analysis was repeated for each node in the context of double knockouts with *MYC* also suppressed in each case (grey bars). (D), *MYC* dependent *TERT* repression and reversal by *AR*. A2780 were transfected with 200 nM non-specific control siRNA (Con), 100 nM *MYC* with 100 nM non-specific (*MYC*), or 100 nM *MYC* and 100 nM each specific siRNA. Cells were harvested after 48 h and RNA extracted for analysis of *TERT* expression normalised to RPS15 by RT-QPCR. Mean ± SEM of *TERT* expression in treated cells relative to control from three experiments (ns: not significant; *: p<0.05; **: p<0.01). (E), Knockdown of *TERT* regulatory transcription factors by RNAi. A2780 were transfected with 100 nM each specific siRNA (RNAi) or non-specific control (NS) and harvested after 48 h. 20 µg protein samples were analysed by western blotting against the respective targets. ERK counter-blots were also performed. Each experiment was performed twice. Representative blots are shown.

Figure 5A shows 4 types of feedforward loop (FFL) motif widely present in transcriptional networks (FFL type I–IV). In each case, two transcription factors (labelled X and Y in the figure) control expression of a third gene, Z. In addition, X also transcriptionally regulates Y. Each interaction may be activating (solid lines) or inhibiting (dashed lines). Of the 8 possible configurations of this motif, type I–IV are designated “coherent”, since the overall effect on Z resulting from the path X→Y→Z is the same as the direct path X→Z in terms of activation or repression. In particular, types I and IV are activating at Z, whereas types II and III are repressive. These have previously been characterised in a kinetic modelling context as delay elements which may reduce noisy expression of Z [75].

It is a reasonable expectation that the expression of a regulated gene Z, such as *TERT*, may be affected by the extent to which multiple FFL centred on its regulation interact and overlap to form coherent activation or repression “modules”, and by the relative dominance of one or other of these subsystems. Specifically, in the current context, we define the “activation module” to be the set of overlapping FFL types I and IV focused on activation of *TERT* and the corresponding repression module to be the set of overlapping FFL types II and III focused on its repression.

We extracted these subsystems from our network model of *TERT* transcription (figure 5B). The method of extraction is detailed in materials and methods and supplemental file Text S1. The activation module comprises 12 nodes including *TERT*, and 31 interactions, of which 22 are stimulatory and 9 inhibitory. *MYC* plays a key role in blocking *TERT* repression in this system through several interactions which antagonise *TERT* repressors. Most other interactions involve *TERT* activators which support each other's expression. The repression module comprises 13 nodes and 27 interactions (9 activating, 18 inhibiting). Therefore, the 2 systems seem reasonably balanced at first glance.

However, further analysis suggests the activation module is structurally more co-operative. Longer paths are present, suggesting more scope for positively reinforcing delays: activation module network diameter (length of the longest of all shortest paths between any 2 nodes in the network) is 3 compared with 2 for the repression module. We also quantified relative participation of each factor in pathways in either subsystem using the betweenness centrality and flow centrality metrics [76], [77]. Betweenness centrality of a node n is the fraction of all shortest paths between all other pairs of nodes in which n participates. By this analysis, *MYC*, *MYCN*, *HIF1A*, *STAT3* and *FOS* all participate strongly as intermediates on shortest paths in the activation module, whereas only *AR* plays this role in the repression module (table 6). Flow centrality is a related measure which considers all paths, not only geodesics. As can be seen from table 6, this analysis provided similar results to the betweenness centrality analysis. Therefore, the *TERT* activator subsystem is more co-operatively connected in our model.

**Table 6:** Node betweenness centrality values for the activation and repression modules.

Subnetwork	Node	Betweenness	Flow Betweenness
AM	*JUN*	0.0000	0.200
	*SP1*	0.0000	0.833
	*MYCN*	0.0545	6.917
	*MXD1*	0.0000	0.533
	*RELA*	0.0000	1.667
	*MYC*	0.0909	10.500
	*HIF1A*	0.0091	1.167
	*FOS*	0.0091	3.750
	*SP3*	0.0000	0.200
	*STAT3*	0.0091	3.750
	*NR2F2*	0.0000	0.200
	*TERT*	0.0000	0.000
RM	*AR*	0.0151	2.500
	*JUN*	0.0000	0.000
	*TP53*	0.0000	0.000
	*SP1*	0.0000	0.825
	*E2F1*	0.0000	0.000
	*MYCN*	0.0000	0.125
	*RELA*	0.0000	0.658
	*MYC*	0.0000	0.125
	*HIF1A*	0.0000	0.458
	*FOS*	0.0000	0.125
	*SP3*	0.0000	0.700
	*STAT3*	0.0000	0.325
	*TERT*	0.0000	0.000

### Robust *MYC* dependent *TERT* repression

To determine the contribution of each factor to overall activation of *TERT* in the model, we investigated the effect of simulating constitutive activation or suppression of each node individually. As above, appropriate changes were made in the rule-tables of each node and steady states were investigated. We quantified, in each case, the total proportion of model states which evolve to steady states with *TERT*-on. The weak components of statespace (basins of attraction) were obtained by depth-first search and the sizes of these were quantified. The total proportion of statespace contributed by each basin of attraction harbouring a *TERT*-on steady state was quantified (figure 5C). In BN models, attractors are usually taken to be distinct “phenotypes” [78]. Hence, the steady states should be interpreted as different network states capable of supporting *TERT* expression. The fraction of statespace associated with them is a rough measure of the probability that any deviation from those steady states (through noise or intervention) will be reversed and maintain *TERT*-on rather than leading to a steady state transition to stable repression.

Most factors had little effect individually on the stable expression of *TERT* in the model. However, constitutive repression of both *MYC* and *FOS* fully ablated all stable on-states (black bars for basal model, absent for *MYC*- and *FOS*-off). Therefore, the frequently observed critical role of *MYC* in *TERT* expression was reproduced [2], [79]. Suppression of the *MYCN* and *HIF1A* nodes also had a substantial impact on *TERT*. *MYCN* suppression produced 2 stable off-states, associated with 63.5% and 5.7% of model states, in addition to a small oscillator (5%). The remaining 25.7% of system states associate to the single remaining on-state. *HIF1A* suppression produced a single large off-state associated to 78.9% of statespace and a single smaller on-state. It is noteworthy that these factors all score as important in the betweenness analysis of the activation module, above.

Among the repressors, constitutive activation of *MXD1* produced the largest off-state (61.6% of statespace). Activation of either *NR2F2* or *TP53* had a mild effect in producing small off-states. Despite its key topological role in the repression module, activation of *AR* did not suppress *TERT*. Instead, constitutive *AR* suppression resulted in an off-state, though its size was negligible (0.7%). Overall, we make 2 conclusions from these results. Firstly, the model reproduces, from a network perspective, well known findings that both *MYC* and *MXD1* are centrally important in *TERT* expression [80], [81]. Secondly, the impact on *TERT* expression of targeting individual activators in the model seems to depend closely on their structural contribution in the activation module.

Because of the known importance of *MYC* in *TERT* regulation in the cellular setting, we next investigated the resistance to reversal of *MYC* dependent *TERT* repression observed in our modelling context. Starting from the *MYC* constitutively suppressed rule set, we again modified the rules for each of the other transcription factor nodes to simulate co-activation or co-repression alongside *MYC* inhibition. Steady states were analysed and the ability of each condition to restore *TERT* activity relative to the *MYC* ablated state was scored (figure 5C, grey bars – presence of a grey bar indicates recovery of *TERT* expression). Most secondary ruleset mutations had no effect. However, *AR* suppression resulted in complete *TERT* recovery, consistent with its central topological role in the repression module. *SP3* suppression was also able to promote partial recovery, producing a single small on state (8.9% of statespace). Therefore, *MYC* dependent *TERT* repression appears substantially robust in the model, but is still not entirely resistant to reversal.

To determine whether this prediction holds experimentally, we conducted RNAi double knockdown experiments in A2780 cells to simultaneously reduce expression of *MYC* alongside individual *TERT* repressors. We examined co-suppression of *MXD1*, *TP53*, *AR*, *SP3*, or *JUN*. Western blotting of each RNAi target following transfection of 100 nM non-specific or specific siRNA in figure 5E demonstrates that each individual siRNA successfully resulted in target knockdown, though the efficiencies varied between siRNA. Control blots for ERK were also performed. In most cases, RNAi had no effect on ERK, although *SP3* knockdown did slightly reduce ERK levels, suggesting positive regulation of ERK by *SP3* in these cells. However, knockdown of *SP3* itself was clearly substantially greater.

We next quantified the effect of the double knockdowns on endogenous *TERT* expression by RT-QPCR in A2780 cells. As expected from its well known role in endogenous *TERT* regulation, *MYC* knockdown alone substantially reduced *TERT* mRNA levels to 40.6% of the non-specific control transfectants. Interestingly, as predicted by the model, *AR* co-suppression substantially restored *TERT* expression relative to *MYC* knockdown alone. *SP3* knockdown had a very mild effect, as predicted, restoring *TERT* expression to 57% of control levels. Additionally, *TP53* knockdown was found to have slightly stronger effect than *SP3*, restoring levels to 62.9% of control, although this was not predicted by the model. Neither *JUN* nor *MXD1* knockdown had a significant effect in our hands. Therefore, our model successfully predicted that *MYC* inhibition produces *TERT* suppression which is largely but not completely resistant to targeted attempts at reversal. Additionally, the model identified the only factor, *AR*, which was able to reverse *MYC* dependent *TERT* suppression by RNAi in these experiments. Therefore, the model can be used to provide previously unknown mechanistic insights into *TERT* regulation.

### Modelling Ets-factor gain of function at the *TERT* gene

During preparation of this manuscript, several papers emerged reporting identification of somatic and germline mutations in the *TERT* promoter in a range of tumour types and cell lines including melanoma, glioma, hepatocellular carcinoma and others [23]–[25]. Among those reported to occur at high frequency, particularly in melanoma, are the C228T and C250T mutations which occur close to the transcriptional start site and introduce gain of function binding sites for Ets family transcription factors resulting in increased *TERT* promoter activity. Since we have not considered Ets-factors in our model, we were interested to determine the effect of adding an Ets-factor into the basal model.

**Figure 6:**
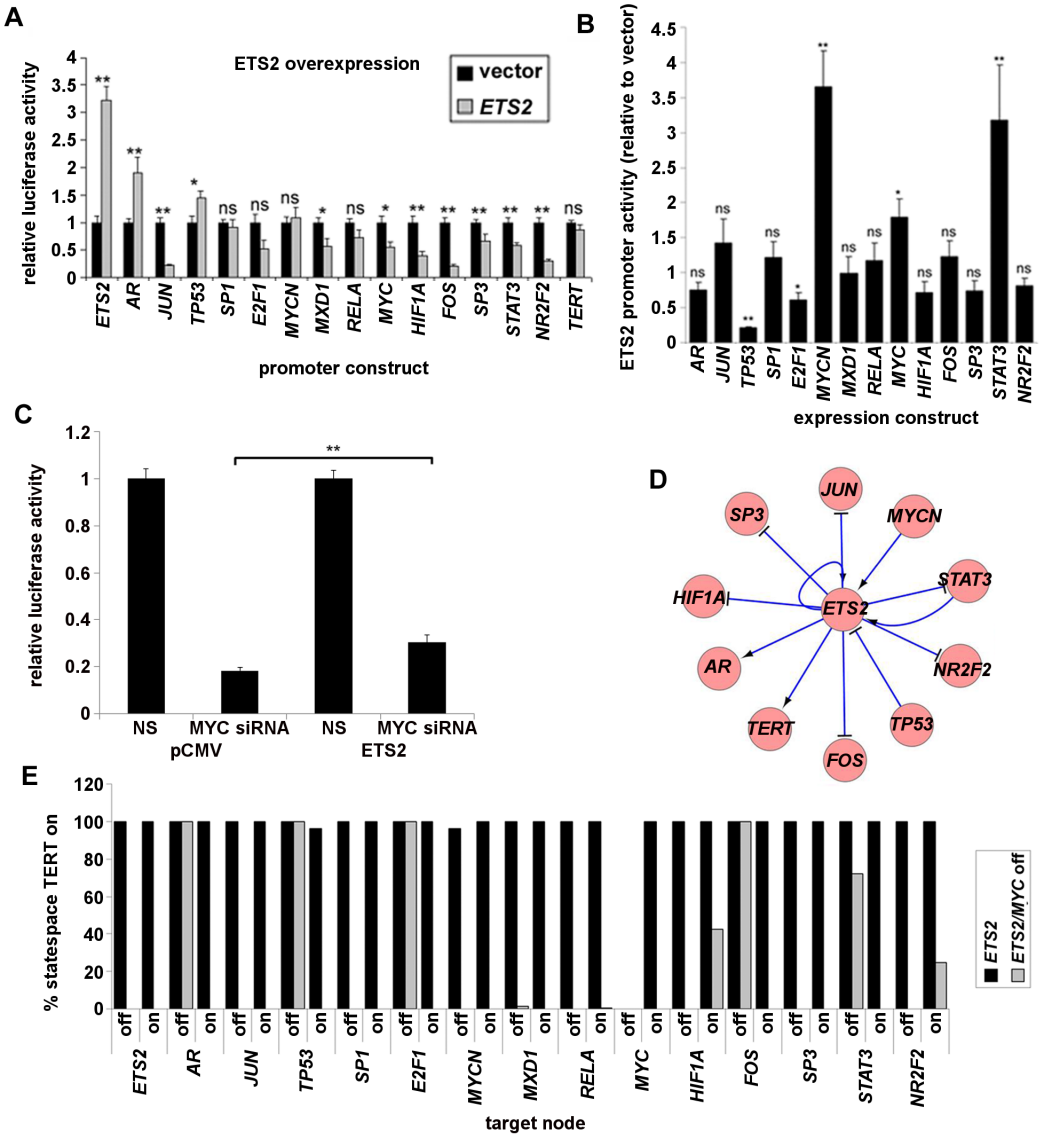
Simulation of Ets family transcription factor gain of function at the *TERT* promoter. (A), overexpression of *ETS2* against the promoter panel. A2780 cells were transfected with the luciferase reporters shown. Each reporter was co-transfected alongside vector control or pCMV-*ETS2*. 48 h post-transfection, promoter activities were analysed by luciferase assay. Mean ± SEM of three experiments (ns: not significant; *: p<0.05; **: p<0.01). (B), regulation of the *ETS2* promoter by the transcription factor panel. A2780 cells were co-transfected with *ETS2*-luciferase reporter alongside vector control or expression vectors shown. 48 h post-transfection, promoter activities were analysed by luciferase assay. Mean ± SEM of three experiments (ns: not significant; *: p<0.05; **: p<0.01). (C), effect of *ETS2* expression on the *TERT* promoter under *MYC* inhibition. A2780 cells were co-transfected with the *TERT*-luciferase reporter and with pCMV control or pCMV-*ETS2* with non-targeting or *MYC*-specific siRNA. 48 h post-transfection, promoter activities were analysed by luciferase assay. Mean ± SEM of three experiments (D), interactions in the *ETS2* subnetwork added into the model with cutoffs FC>1.5, p<0.01 from the transfection data. The subnetwork was visualised in Cytoscape [105]. Arrows indicate activation, T-shape indicates repression. (E), Effect of single- and double-node targeting on *TERT* on-states in the *ETS2* modified model. Rule-sets for each node were modified in turn individually (black bars) to simulate constitutive repression or activation. For each rule-set change, statespace was derived and the proportion of system states evolving to attractor states with *TERT* stably on was quantified. The analysis was repeated in the background of the *MYC* suppressed rule-set (grey bars).

We therefore acquired a promoter reporter and expression vector for *ETS2* and tested these as before for the effect of *ETS2* overexpression in our promoter panel and for the effect of our expression vector panel on the *ETS2* promoter (figure 6A and 6B). The *ETS2* promoter was suppressed by overexpression of *TP53* and activated by *MYCN* and *STAT3* in addition to its own overexpression (figure 6B). Overexpressed *ETS2* up-regulated its own promoter and that of *AR*, and repressed those of *JUN*, *HIF1A*, *FOS*, *SP3*, *STAT3* and *NR2F2* with fold change >1.5 and p<0.01 which satisfied our cut-offs described earlier. We did not detect a significant effect of *ETS2* overexpression alone on our *TERT* promoter construct. However, we were also interested in whether there may be interplay between *MYC* and *ETS2*. We therefore co-transfected cells with *MYC* siRNA and *ETS2* expression vectors (figure 6C). *MYC* siRNA strongly repressed promoter activity to 18% of control levels in vector co-transfected cells. However, in *ETS2* co-transfected cells, promoter activity was only reduced to 30% of control levels. Hence, *ETS2* did appear to stimulate the *TERT* promoter under conditions of *MYC* inhibition. It is possible that c-Myc protein at the *TERT* promoter participates in complexes that occlude Ets2 binding. Since *ETS2* expression did stimulate the promoter in these conditions and our objective is to model a gain of function, we included *ETS2* as an activator of *TERT*. These interactions were incorporated into the model to generate a new 16 node network. The included *ETS2* subnetwork is shown in figure 6D.

We re-performed the analysis of the effect of constitutive activation or suppression of each node under the new basal 16-node *ETS2*-added condition (figure 6D, black bars). In this analysis, *TERT* repression was still achieved by *MYC* inhibition. However, this was the only modification able to substantially ablate *TERT* on-states in the new model. Loss of *MYCN* or activation of *TP53* were the only other modifications to have any effect, though these effects were very mild. In contrast to the results in figure 5C, when we repeated the analysis under the condition of *MYC* inhibition in the *ETS2*-added model (figure 6D, grey bars), *TERT* repression was found to be fragile and easily reversible, rather than robust. This is in line with our finding that *ETS2* overexpression stimulated the *TERT* promoter only when *MYC* was knocked down, although the model did not report that *ETS2* itself recovered *TERT* expression. Rather, not only loss of *AR*, but also of repressors *TP53* and *E2F1* or activation of *HIF1A* caused complete or substantial recovery. Therefore, *ETS2* gain of function may enhance *TERT* expression stability. Surprisingly, recovery was also observed when activators *FOS* and *STAT3* were suppressed, or when repressor *NR2F2* was activated. These effects presumably result from reprogramming of the network balance through introduction of *ETS2* interactions at a variety of nodes (figure 6D).

### Topological control of *TERT* on state multiplicity in the model

Our results so far suggest that the model can predict at least some experimentally verifiable aspects of *TERT* regulation. To explore in more detail the role of the transcriptional neighbourhood topology in *TERT* regulation, we focused on the roles of the activation and repression modules (AM/RM) in the model by specifically targeting their connections.

One problem in analysis of the role of topology in complex networks is that reagents are unavailable to efficiently target multiple individual network interactions in a selective manner. However, panels, or ensembles, of random Boolean networks have previously been used effectively as a statistical approach to probe how topological features or interventions regulate system dynamics [82], [83]. We have adopted this approach to study to study the roles of AM/RM. We are unaware of any BN study that has previously investigated roles of these feedforward systems in network dynamics. AM/RM edges were deleted from the model in a series of random attacks of increasing severity targeting either the AM alone or both AM/RM. Probability for each edge to be deleted was varied between 0.1 and 0.7 in increments of 0.05. This produced a series of re-wired networks based on the original model but with differing contributions from AM/RM. All edges connecting directly to *TERT* were left unmodified in each case.

**Figure 7:**
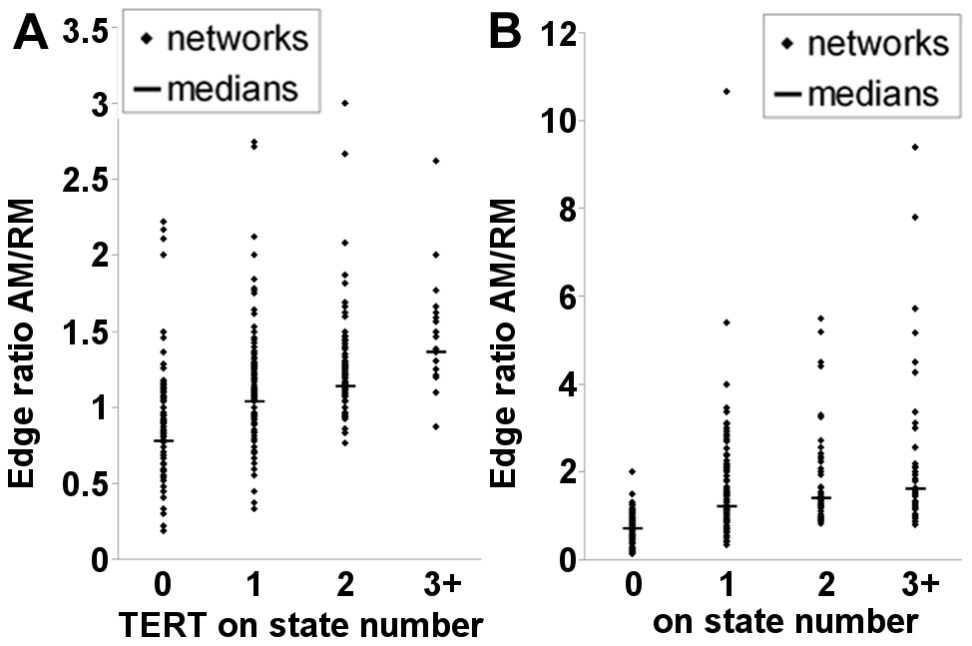
Topological control of *TERT* on-state multiplicity in the model. (A), influence of activation module dominance on *TERT* on-state multiplicity. Topology of the model was altered by a series of 600 random attacks deleting activation and repression module interactions with increasing probability. Direct interactions with *TERT* were left unaltered in all attacks. The remaining sub-networks were extracted from each model variant as described in materials and methods and the number of edges in each were counted to determine the edge ratio AM/RM. The statespace of each model was calculated and the number of stable on states present for the *TERT* node was quantified and plotted against the calculated AM/RM edge ratio for each variant network. Significance of edge ratio population differences was tested in Matlab by Wilcoxon rank-sum test (**: p<0.01). (B), influence of AM dominance in random networks. A series of 300 (15 node) networks was generated with semi-random edge seeding and increasing edge density. All networks were constrained to have one regulated node which was connected downstream of all others. The number of activators and repressors of the node was allowed to vary randomly. Statespace and AM/RM edge ratios were calculated for each network and compared as in (A), calculating number of stable on-states for the fully connected node. Significance of edge ratio population differences was tested in Matlab by Wilcoxon rank-sum test (**: p<0.01).

We extracted AM/RM from each resulting modified network and quantified the number of edges remaining in the re-wired subnetworks in each case as a measure of overall prevalence of either system. In the case of the “wild-type” basal model, AM is slightly dominant with an edge ratio AM/RM of 1.15 although, as noted, the AM also has greater betweenness. We also calculated the statespace of each model variant and quantified the number of *TERT* stable on-states. The relation between these metrics is shown in figure 7A. It is clear that AM dominance associates with emergence of multiple on-states. The median edge ratios for 0, 1, 2, or 3 or more on-states were 0.78, 1.04, 1.14 and 1.36, respectively. These differences were all highly significant (p<0.01, by Wilcoxon Rank Sum).

Lastly we investigated the roles of AM/RM in the more general case of semi-random networks. We generated 300 (15 node) networks of increasing edge seeding density (100 networks each with each possible edge included with probability 0.1, 0.15, or 0.2). The probability for any included edge to be activating or inhibitory was 0.5. We imposed the constraint that the 15^th^ node was downstream of all other nodes as in the case of *TERT* in our main model. Each node was randomised to be activating or inhibitory at node 15. Hence, this represents the scenario where a wider range of potential topologies for AM/RM are possible than in the case of our deletion analysis above. Figure 7B confirms that association between relative dominance of the AM feedforward substructure and on-state multiplicity for the regulated node was also retained in this second network panel.

Our results overall provide an insight into the roles of these systems in BN models and suggest that a co-operative activation module may provide redundancy in respect of the individual activators of a regulated gene which is its focus, consistent with the proposed functions of FFL type I and IV. In the context of *TERT* regulation, this could allow for multiple states which are permissive for *TERT* expression. This may have potentially important implications for strategies targeting the expression of *TERT* in cancer cells since telomerase inhibition by targeting *TERT* activators may be more problematic than was previously thought.

## Discussion

Previous studies have taken mathematical modelling approaches to investigate cellular senescence responses to telomere homeostasis and oxidative stress [84], [85]. The current report is the first investigation of *TERT* transcription using modelling to probe its regulation at the systems level. Telomerase regulation has been widely studied at the level of identification of individual transcription factor regulators of *TERT*. However, like most complex biochemical systems, a clear view of their co-operative activities is lacking. Multiple pathways regulate *TERT* and some such as *MYC* may be more critical than others globally or context-independently, whereas some may play essential context-dependent roles [2], [4], [6], [23], [29], [79]–[81], [86]. From the point of view of therapeutics based around *TERT* transcription, including signalling inhibitors, better understanding of system behaviour would be advantageous. Clearly, endogenous gene transcription involves multiple complex events which are mechanical in nature as well as dynamical, and which are not easily captured in simplistic models. These include chromatin effects, complex formations, promoter melting, abortive initiation, promoter escape and elongation [87]. Classical Boolean networks offer only a qualitative modelling solution and suffer from several major disadvantages. For example, it is necessary to define a static network structure by literature-curation or, as in the model reported here, by experiment. This “snap-shot” does not take account of the possibility that network structure also changes in a dynamic way. This is potentially an extremely interesting route for refinement in future models by combining time-dependent network inference with advanced dynamic features such as memory [88], [89]. However, as an entry point to systems level study of *TERT*, even simple models such as that presented here could be useful. In this study, as a direct consequence of application of the model, a previously unknown role for *AR* in stabilising *MYC* dependent *TERT* repression was revealed. Furthermore, our model predicted the effects of several signal transduction inhibitors, including the powerful *TERT* repressor effect of SU6656 in cells. The model also suggests that Ets-factors may functionally co-operate with *MYC* expression to enhance *TERT* transcription stability.

It is noteworthy that the experiments required in order to show phenotypic effects of telomerase inhibition are usually very long term and their outcome is rarely certain at the start of an experiment. In our previous work, BIO was effective in suppressing *TERT* expression but not in long term treatments [4]. Our model suggests this may be the result of susceptibility to noise, which is in line with our previous observations. Therefore, the possibility that small reporter screens as performed for SU6656, coupled with application of models, could be used as a filter to increase confidence in the potential for the long term effects of a compound is intriguing. Modelling might also assist in clarifying which network interventions are fragile or reversible in targeting telomerase, potentially allowing better approaches to be designed. To develop our network model we applied a screening approach. To our knowledge this is the first report of a BN model of transcription generated in this way. We initially tested a range of literature-curated models which did not perform as well as the data derived model. This may be because the interactions in these networks are curated from a range of experiments performed in different contexts. The overlap between our screening results and the literature was highly significant, although some interactions were found to be non-concordant. These may reflect different effects in different cell systems or the use of different constructs. Regulation of transcriptional network interactions is likely to differ substantially among different cell lines. In contrast, excellent models of core signal transduction pathways can be literature curated since specific cell models and relatively standard reagents are often widely employed in studying their kinetics. That the model was able to make several de novo predictions that could be verified in cells suggests that this might be a useful general approach to develop BN models of transcriptional systems.

Because of the nature of the threshold rule, which is a balance between activating and repressive interactions, in model development we felt that a network having an evenly balanced number of *TERT* activators and repressors would likely give better results. In preference to reducing the model size to achieve that balance, we therefore retained *STAT3* as an activator throughout model selection. We previously found that STAT3 protein binds the core TERT promoter and we repeated this analysis here. As discussed in supplemental file Text S1, a model without *STAT3* still had most of the behaviour of the complete model.

One limitation of the Boolean framework analysis is in modelling constitutive repression or over-expression by mutating the rules for a node to be “always off” or “always on”. This does not reflect the biological setting, where a relative decrease or increase in concentrations would more likely be observed. One possibility to refine the existing model would be to make use of the thresholds and weights for nodes and interactions to more accurately reflect this scenario. However, our approach did capture behaviours that we demonstrated experimentally. Thus, the current qualitative model does seem able to reproduce some essential aspects of endogenous *TERT* regulation.

Although *MYC* has been well studied in the context of *TERT* regulation in cancer cells, the stability of *TERT* suppression by *MYC* inhibition is not well understood. Our modelling results suggested this state is substantially but not completely resistant to reversal. Our structural analysis of the model suggests that *MYC* may be important not only in its direct effects at the *TERT* promoter but also by its “brokerage” role throughout the transcription network.

We examined the role of network topology in promoting stable *TERT* expression in the modelling context. We identified feedforward systems in the network whose structure suggests coordinated functional regulation of transcriptional interactions. The activation module sub-system was topologically dominant over the repression module sub-system in the “wild-type” model. Targeted edge attacks showed the activation module contributes to *TERT* on state multiplicity in the model. If this model prediction is also similar to the endogenous scenario, then network organisation would indeed play a significant role in *TERT* transcriptional stability.

Clearly, these results are an untested extrapolation, but interesting nevertheless since experimental confirmation that individual interactions give rise to emergent behaviour would require reagents capable of ablating individual endogenous interactions. No current reagents are able to efficiently produce this effect since reagents such as siRNA can only target gene products and not their interactions. On the other hand, BN panels have previously been used to infer relations between topology and network dynamics [82], [83]. To our knowledge, the roles of the feedforward systems we describe have not been directly tested before and our results suggest they may play important general roles in network dynamics. In our model of *TERT* regulation, the activation module was more co-operative than the repression module by the measures of betweenness centrality and flow centrality (table 6).

In the endogenous context, *TERT* expression clearly is essential in most cancer cells and it is likely that a variety of backup mechanisms exist which are capable of subverting *TERT* suppression as we previously observed [4]. A highly co-operative AM could promote stable expression dynamics of *TERT* by providing for long signal delays and substantial redundancy among activators, thereby allowing the transcriptional neighbourhood to adopt divergent states while maintaining expression of *TERT* and promoting stability.

In respect of the therapeutic scenario, multiple approaches have been suggested to target telomerase including transcriptional suppression through intervention with its regulatory pathways [86], [90], [91]. If this is indeed a viable approach, and if the true regulatory network does indeed share the features we describe, then it seems one should aim to hit the pathways as broadly as is possible while retaining acceptable toxicity. Suppression of *TERT* through *MYC* knockdown was substantially robust but, as we have shown, also fully reversible under appropriate conditions. Hence, it is probably insufficient to consider targeting *TERT* transcription in terms of individual factors since targeting any single transcription factor may not guarantee repression in the long term. Advanced therapeutic approaches based on *TERT* suppression may need to explicitly target the systems complexity of telomerase regulation by, for example, polypharmacology strategies. In general, broad inhibition of the activation module or broad activation of the repression module may be preferable.

SU6656 appears to engage the latter mechanism and to cause both profound *TERT* suppression in cells and a powerful cell cycle arrest. Given the functions of several repression module factors in cell cycle arrest, DNA damage and senescence, it may be that its co-ordinate activation selectively promotes cell cycle arrest and/or premature senescence rather than a classic telomerase inhibitor phenotype. Similar results might also be obtained with other compounds which engage senescence pathways [92]. If senescence and immortalisation are considered to be opposing processes, regulated by opposing mechanisms, then just as the activation module appears evolved for efficient telomerase expression in immortal cancer cells, it may be that the opposing repression module is evolved for efficiency of arrest. Highly co-ordinated regulation of telomerase expression appears hard-wired in both processes.

## Materials and Methods

### Cell lines, plasmids, siRNA and inhibitors

The cells used were A2780 ovarian adenocarcinoma cells. Reporter pGL3-*TERT* contains the *TERT* promoter region −585/−9, relative to the translational start site. All other reporters were obtained from Switchgear Genomics (Menlo Park, CA). Constructs were *AR* (S7148912), *JUN* (S721598), *TP53* (S721662), *SP1* (S722903), *E2F1* (S719961), *MYCN* (S719321), *MXD1* (S708803), p65 (S720207), *MYC* (S719565), *HIF1A* (S721637), *FOS* (S721638), *SP3* (S711215), *STAT3* (S706087), *NR2F2* (S708524), *ETS2* (S719772). pCMV expression vectors for *MYCN*, *FOS*, *NR2F2*, *STAT3* and *MXD1* were obtained from Cambridge Bioscience (Cambridge, UK). Expression vector for *TP53* was obtained from Clontech Laboratories (Mountain View, CA). We previously reported the expression vectors for *SP1*, *SP3* and HIF1A [29], [93], [94]. Expression vectors for *E2F1* and *RELA* [95], [96] were kind gifts from Professor Kevin Ryan (Beatson Institute for Cancer Research, Glasgow, UK). Avian *MYC* and v-Jun vectors [97], [98] were kind gifts from Professor David Gillespie (Beatson Institute for Cancer Research, Glasgow, UK). *AR* expression vector [99] was a kind gift from Professor Hing Leung (Beatson Institute for Cancer Research, Glasgow, UK). FR180204, 6-bromoindirubin-3′-oxime (BIO) and SU6656 were obtained from EMD Biosciences (Nottingham, UK). OnTargetPlus siRNA smart-pools against *MYC* (L-003283), *AR* (L-003400), *TP53* (L-003329), *MXD1* (L-009325), *JUN* (L-003268), or *SP3* (L-023096), were obtained from ThermoFisher Scientific (Leicestershire, UK).

### Transfections and luciferase assay

All transfections were performed in triplicate (3 technical replicates) using superfect reagent according to the manufacturer's instructions using a 2.5∶1 ratio reagent∶DNA (Qiagen, Crawley, UK). 250 ng reporter plasmid and expression vector per well were transfected in 96-well luminometer plates (ThermoFisher Scientific UK, Leicestershire, UK). 48 h post-transfection, luciferase activities were determined using luciferase assay reagents according to the manufacturer's instructions (Promega Ltd, Madison, WI). All experiments were repeated at least 3 times (3 biological replicates).

### Western blotting

Protein extracts (1 technical replicate per experiment) were prepared in passive lysis buffer (Promega Ltd, Madison, WI). Protein concentrations were estimated at OD595 using the BioRad protein assay (BioRad Laboratories Ltd, Hemel Hempstead, UK). 20 µg protein were separated by SDS-PAGE, blotted onto PVDF filter (Millipore, Watford, UK) and blocked overnight in PBS-T containing 5% non-fat dried milk. Antibodies used were Androgen Receptor (ab9474) and *TP53* (ab7757), obtained from Abcam (Cambridge, UK), *MXD1* (sc-222), *SP3* (sc-644), *MYC* (sc-764), obtained from Santa Cruz Biotechnology Inc (Heidelberg, Germany) and *JUN* (9165), obtained from New England Biolabs UK (Hitchin, UK) Primary antibodies were detected with HRP-conjugated secondary. HRP was detected using ECL HRP detection reagents (Amersham Pharmacia, Buckinghamshire, UK). All experiments were performed at least twice (2 biological replicates).

### Quantitative RT PCR

Q-PCR was performed in triplicate (3 technical replicates) using Genetic Research Instrumentation (Essex, UK) Opticon monitor equipment and software. Sybr green was used as fluorophore. The primers used were: RPS15, 5′-TTCCGCAAGTTCACCTACC and 5′-CGGGCCGGCCATGCTTTACG; *TERT* 5′-CTGCTGCGCACGTGGGAAGC and 5′-GGACACCTGGCGGAAGGAG. Optical read temperatures were optimised to exclude primer dimers. All treatments were repeated three times (3 biological replicates) and Q-PCR was performed twice for each assay.

### Boolean networks modelling framework and topology analysis

BN comprise a set of N nodes and the set of their activating or inhibitory interactions. Each node takes one of 2 states: 1 (on) or 0 (off). Hence, there are 2^N^ possible combinations of node states. The system is initialised at time t_0_ under some combination of on/off states and the dynamics of each node during simulation of further time steps are determined entirely by a rule-table specifying the next state of each node given each possible state combination of its regulators [100]. In the classical BN all nodes are synchronously updated. We use the “threshold” rule,
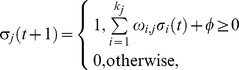
Here, σ_j_(t+1) is the next state of node j, σ_i_(t) defines the current state of the i-th node [90], ω_i,j_ is the weight of the interaction from node i to node j (1 if the interaction is activating, −1 if repressive), and k_j_ is the input-degree of the regulated node j. The threshold variable φ is zero in all rule-sets in this study. Hence, a node will be turned off at time t+1 only in the case that more of its repressors than activators are on at time t. Topology of the basal model was defined using data from the transfection screen. Cut-offs for assignment of an interaction were fold-change in reporter activity, FC>1.5 (up- or down-regulation), and p-value (ANOVA)<0.01. Each ω_i,j_ = 1 if promoter j was activated by transcription factor i, or ω_i,j_ = −1 in the case of repression.

In at most 2^N^+1 time steps during simulation of classical BN, a previous state must be revisited as must all subsequent steps. The system is then locked in an attractor. Commonly, far fewer steps are required. Therefore, BN rapidly converge either to point attractors or limit cycles [101], though we use the informal terminology steady states or oscillations here. Characterisation of these is a principal method for analysis of BN models. In this paper we have performed statespace analysis of all models [73], [102]. We use the brute force approach of calculating each state transition since the networks are small, rather than the reverse approach of computing pre-images [102]. Basins of attraction were identified as the weak components of statespace and the corresponding attractors as their input degree core. The states of the *TERT* node were analysed in each attractor. Limit cycles were considered to have stable *TERT* activity if the node was on in all sub-states. During the study, we developed a Windows console application which implements the statespace analysis and other functions on Pajek net files (http://sourceforge.net/projects/statespaceminer).

Topological analysis of models was performed in Pajek [103], UCINET [104] and Cytoscape [105]. To extract AM and RM, firstly loops were removed and a tripartite partition P placed on the nodes on the basis of their relation to *TERT*: P_1_, *TERT* repressors; P_2_, *TERT* activators; P_3_, *TERT*. For extraction of FFL type I and IV (the activation module, AM), 4 edge sets (E_1–4_) were extracted from the partitioned network:
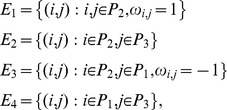
such that E_1_ contains all positive interactions among *TERT* activators, E_2_ contains the edges between *TERT* activators and *TERT* itself (by definition, ω_i,j_ = 1), E_3_ contains inhibitory edges from *TERT* activators to repressors, and E_4_ contains the edges between *TERT* repressors and *TERT* itself (by definition, ω_i,j_ = −1). Common union of edge sets was performed. Since each node connects with *TERT*, network reduction by elimination of nodes with degree less than 2 and their associated edges ensures recovery of an overlapping system of complete triads comprising FFL type I and IV. The repression module (RM) was similarly defined to comprise overlapping FFL types II and III. A pseudocode description of the extraction algorithms using the network adjacency matrix is given in supplementary file Text S1. In comparing relative dominance of each subnetwork, we use the simple metric of the edge ratio AM/RM.

### Literature search

We apologise to any authors if we have overlooked your study which meets our inclusion/exclusion criteria in the preparation of table 4. Please contact us and the relevant references will be included in any future publications concerning this model or refined versions. To determine the literature in this area, we analysed references within the MetaCore database [4], the Transcriptional Regulatory Element Database [106], in addition to performing PubMed searches. In our PubMed searches, we included a range of search terms to find regulator → target interactions including ‘“regulator” AND “target promoter”’, ‘“regulator” AND “target mRNA”’, ‘“regulator bind*” AND “target promoter”’, ‘“regulator represses target”’, ‘“regulator activates target”’, and similar variations. We tested both gene and common protein names and, in the case of regulators which are intrinsically parts of a complex such as NF-κB, we also tested the name of the complex. In the model we consider only regulator → target transcriptional interactions. We therefore applied the following inclusion/exclusion criteria on the searches:

Inclusion criteria

1. Demonstration in human cells and any or all of:
2. Selective modulation of regulator causes regulation of target mRNA.
3. Selective modulation of regulator causes regulation of target promoter activity.
4. Regulator binds to target promoter in vitro or in vivo with effect on target mRNA or protein

Exclusion criteria

1. Only found in non-human system.
2. Regulator binds target promoter but without validated expression change.
3. Genomic-scale studies without specific validation of effect on target.
4. Regulator and target proteins physically and functionally interact to regulate a third gene but without evidence of direct regulator → target interaction as defined by the inclusion criteria.

### Statistical analysis

Statistical analysis of all transfection and QPCR experiments was performed by one way ANOVA or Student's t-test in Microsoft Excel. Analysis of distribution of AM/RM edge ratios was performed by Wilcoxon Rank-Sum using Matlab.

## Supporting Information

Text S1 **Detail of model development.**
(DOC)
