# Supplementary material for: Mathematical Model of a Telomerase Transcriptional Regulatory Network Developed by Cell-Based Screening: Analysis of Inhibitor Effects and Telomerase Expression Mechanisms
Source: PLoS Comput Biol. 2014 Feb 13;10(2):e1003448. doi: 10.1371/journal.pcbi.1003448 (PMC3923661; doi:10.1371/journal.pcbi.1003448)
Supplement: Text S1 — Detail of model development. (DOC) [file pcbi.1003448.s001.doc]

# Generation of the model

We originally intended to develop a model to explain *TERT* suppression by the GSK3 inhibitor BIO and potentially to explore oscillations in *JUN* and *TERT* expression under BIO treatment which were observed in our previous publication (Bilsland et al., [PLoS One.](http://www.ncbi.nlm.nih.gov/pubmed/19649288) 2009 Jul 31; 4(7):e6459), cited in the main text. We began with a version of the transcriptional network inferred from microarray data in treated A2780 cells, which we reported in that paper (Figure S1). The network is based on curated literature-reported interactions and was inferred using the MetaCore GeneGo platform with a custom designed workflow described in the publication. Note that nomenclature of the MetaCore network objects differs from gene nomenclature convention. We use attractor states to show the behaviour of models where red signifies on green signifies off.


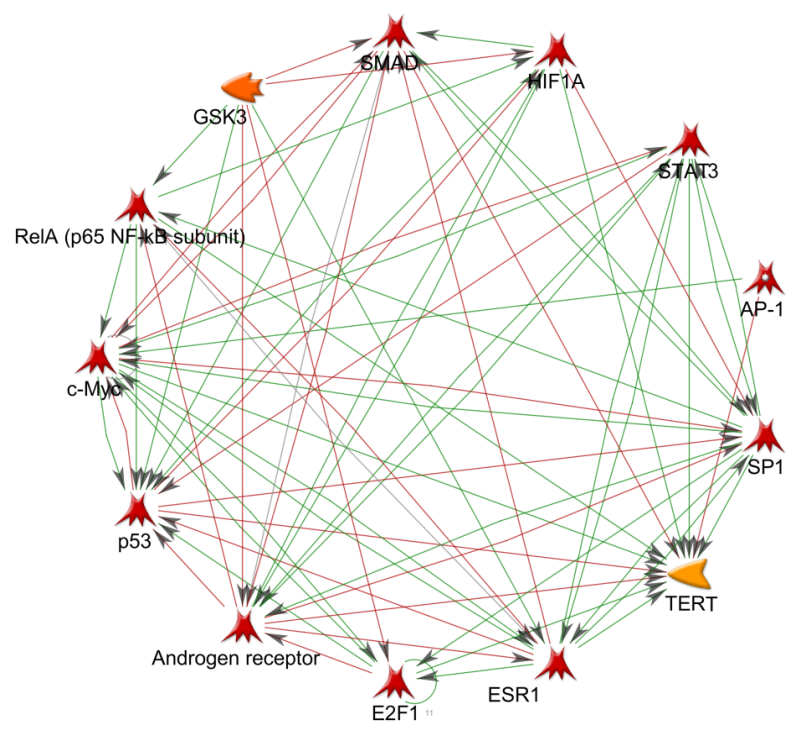


**Figure S1: Literature-derived model from MetaCore. Green arrows indicate activation effects, red arrows indicate repression.**

As detailed in materials and methods, we applied the threshold rule to this topology and examined attractor states of the model. We chose the following criteria for model selection:

1. At least one attractor state with *TERT* stably on should exist and should associate with a significant part of model statespace. Limit cycles were allowed if *TERT* was on in all sub-states.
2. We specified that inhibition of *MYC* in the model should result in decrease in the number of *TERT*-on attractor states or a decrease in the total fraction of statespace associated with them.
3. Modelling the effect of GSK3 inhibition should produce *TERT* inhibition. We were interested in the possible emergence of oscillations but this was not a requirement.

The first literature model had a single steady state with *TERT*-on, shown in figure S2A. We simulated *MYC* inhibition by setting all rules in the *MYC* rule-table to 0 to produce constitutive repression. This resulted in a single attractor, also with *TERT*-on (figure S2B). Therefore, in this model, simulating *MYC* inhibition did not result in *TERT* suppression. We simulated GSK3B inhibition as for *MYC*, setting all rules for the GSK3B node to 0. This also resulted in a single attractor state, also with *TERT*-on (figure S2C). Therefore, simulating GSK3B inhibition did not suppress *TERT* in this model and the model was rejected.


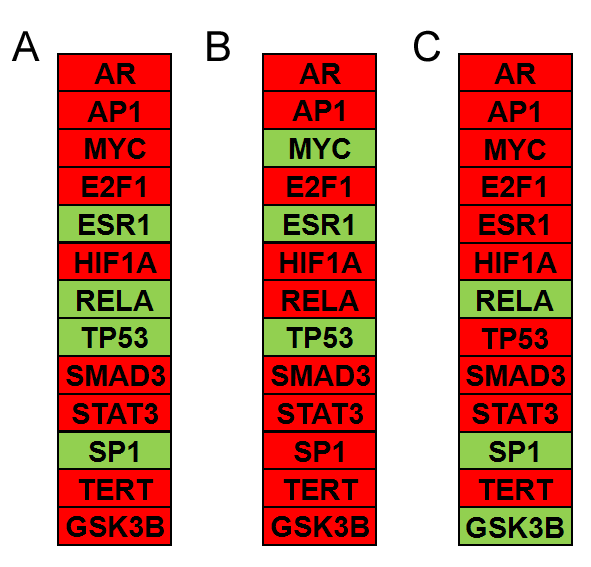


**Figure S2: Attractor states of the model based on the literature inferred network from Bilsland et al.,** [**PLoS One.**](http://www.ncbi.nlm.nih.gov/pubmed/19649288) **2009 Jul 31; 4(7):e6459. (A) Basal conditions, (B) *MYC* inhibited, (C) GSK3B inhibited. Red indicates node was on in the attractor, green indicates off.**

We next examined an expanded version of the literature based model, incorporating more transcription factors known to affect *TERT* expression (figure S3). The basal model had 2 attractor states, both with *TERT*-off and was not investigated further (figure S4).


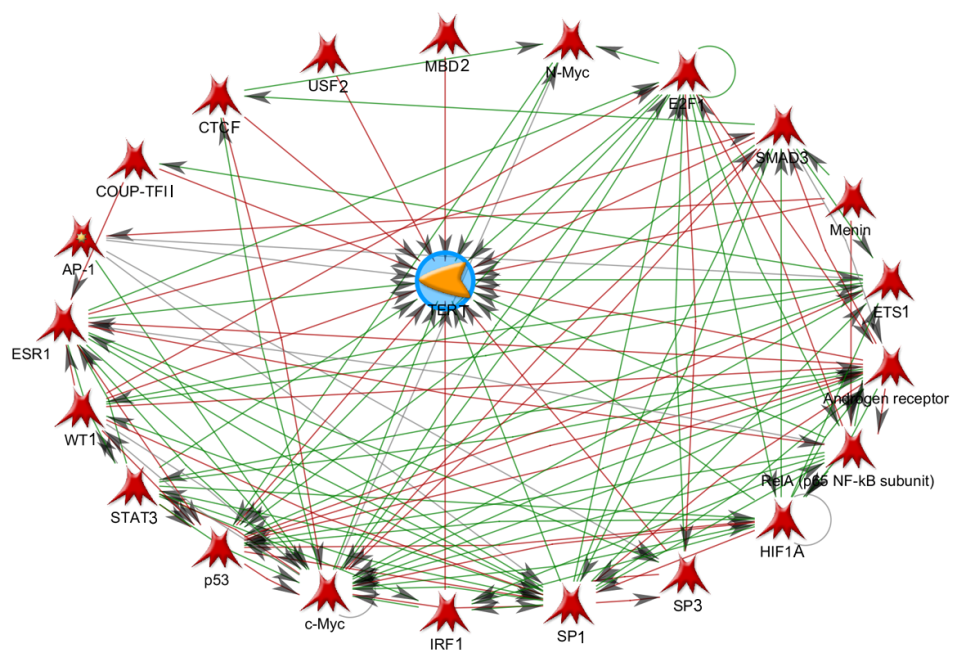


**Figure S3: Expanded literature-derived model from MetaCore. Green arrows indicate activation effects, red arrows indicate repression.**


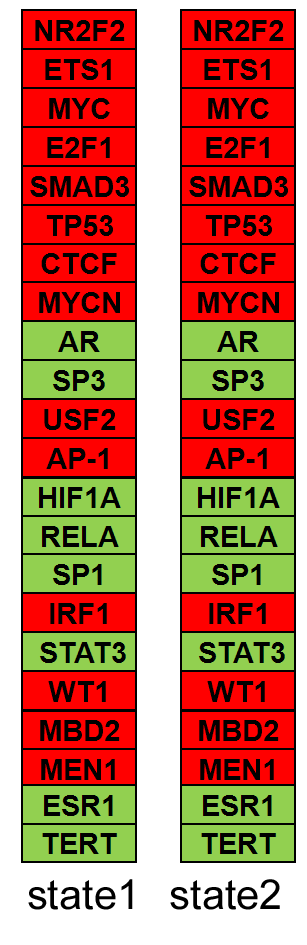


**Figure S4: Basal attractor states of the expanded model. Neither state had TERT-on and the model was not investigated further.**

Given the deficiencies of these modelling attempts, we then devised the transfection screening strategy to define the *TERT* transcriptional network in the A2780 cell line. As described, we obtained both promoter-reporter and expression vector constructs for 14 transcription factors. We performed transfection screening and tested several values of fold-change (FC) and p-value (ANOVA) as cut-offs for an interaction from the screen to be included in the model (table S1). Additionally, we performed BIO treatment against the promoter panel in order to determine a set of rule changes to model the effect of GSK3 inhibition. The threshold rules were applied to each network and the resulting models evaluated.

| FC | p |
| --- | --- |
| >2 | <0.01 |
| >2 | <0.05 |
| >1.5 | <0.01 |
| >1.5 | <0.05 |
| n/a | <0.01 |
| n/a | <0.05 |

**Table S1: Cut-off values tested for interaction assignment.**

A version of the model with FC>2, p<0.05 had 78 interactions. It should be noted that to retain all *TERT* regulators, it was necessary to relax the cut-offs for several TF->*TERT* interactions which did not achieve FC >2 (*JUN*, *MYC*, *FOS*, *SP3*, *STAT3*). These are all well-known regulators of *TERT* and therefore, their exclusion from the model would have considerably negated the purpose of the study. In particular, inclusion of *MYC* is necessary according to our selection criteria. *STAT3* did not achieve the significance threshold but was initially left in since it is a known *TERT* regulator (see citations below and in the text – a fuller account of the role of *STAT3* is given below). The 2-fold requirement was applied to all other interactions.

This model had 3 basal attractor states, all with *TERT*-on (figure S5A). State 1 was the largest, associated to 74% of the model statespace. State 3 was negligible, associated to 0.2% of the statespace. Simulating *MYC* inhibition also resulted in 3 states, 2 with *TERT*-off and 1 with *TERT*-on (figure S5B). These were identical in their share of statespace to the corresponding basal states. Hence, *MYC*-off state 1 was the largest and also had *TERT*-off. Therefore, the model passed the second criterion. The effect of BIO on the promoter panel under these cut-offs was activation of both *E2F1* and *STAT3*. Modelling this effect resulted in the 2 states shown in S5C. The first state, accounting for 96.9% of the statespace, has *TERT*-on. The smaller state 2 has *TERT*-off. Hence, this variant did predict a marginal TERT repression by BIO and weakly passed the third criterion. However, as noted, it was necessary to relax cut-offs for the effect of several factors at the *TERT* promoter and so other models were also investigated.

**
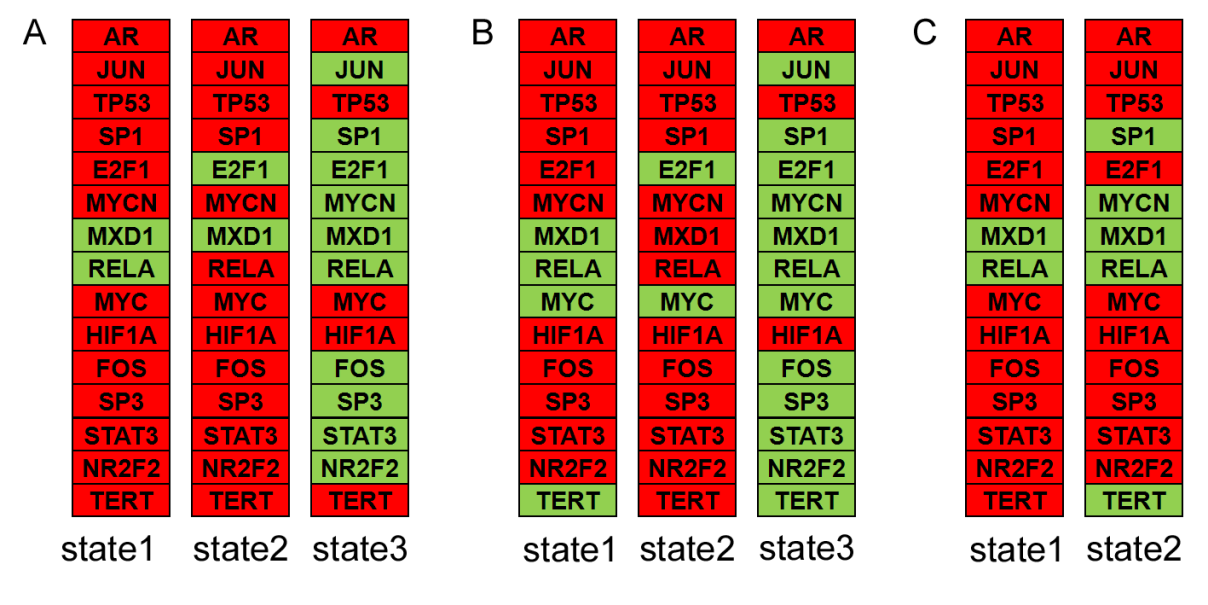
**

**Figure S5: Attractor states of the model based on the cut-offs FC>2, p<0.05. (A) Basal conditions, (B) *MYC-*inhibited, (C) BIO simulation.**

Using FC > 2, p < 0.01 we obtained 67 interactions. The basal model had 4 attractor states (figure S6A). The largest of these was state 1, with *TERT*-off, occupying a basin of attraction comprising 75.4% of statespace. Hence, most states of this model transition to a state of *TERT* suppression, so the model failed our first criterion. Basal state 3 occupies a negligible basin of attraction comprising only 0.2% of the statespace. Simulating *MYC* inhibition resulted in 3 states (figure S6B). It can be seen that an additional *TERT*-off state was present. However, this results directly from conversion of the very small basal state 3. State 1 under *MYC* suppression is identical in its share of statespace with basal state 1. The model is thus a very weak pass on criterion 2 on the basis of the gain of a very small additional off-state. However, the gain is negligible in reality. The effect of BIO under these cut-offs was activation of *STAT3* only. The resulting states were identical with the basal states 1, 2, and 4 in S6A and are not shown separately. However, the *TERT*-off state 1 was reduced in its share of statespace. Hence, this model did not predict *TERT* suppression by GSK3 inhibition and failed criterion 3.


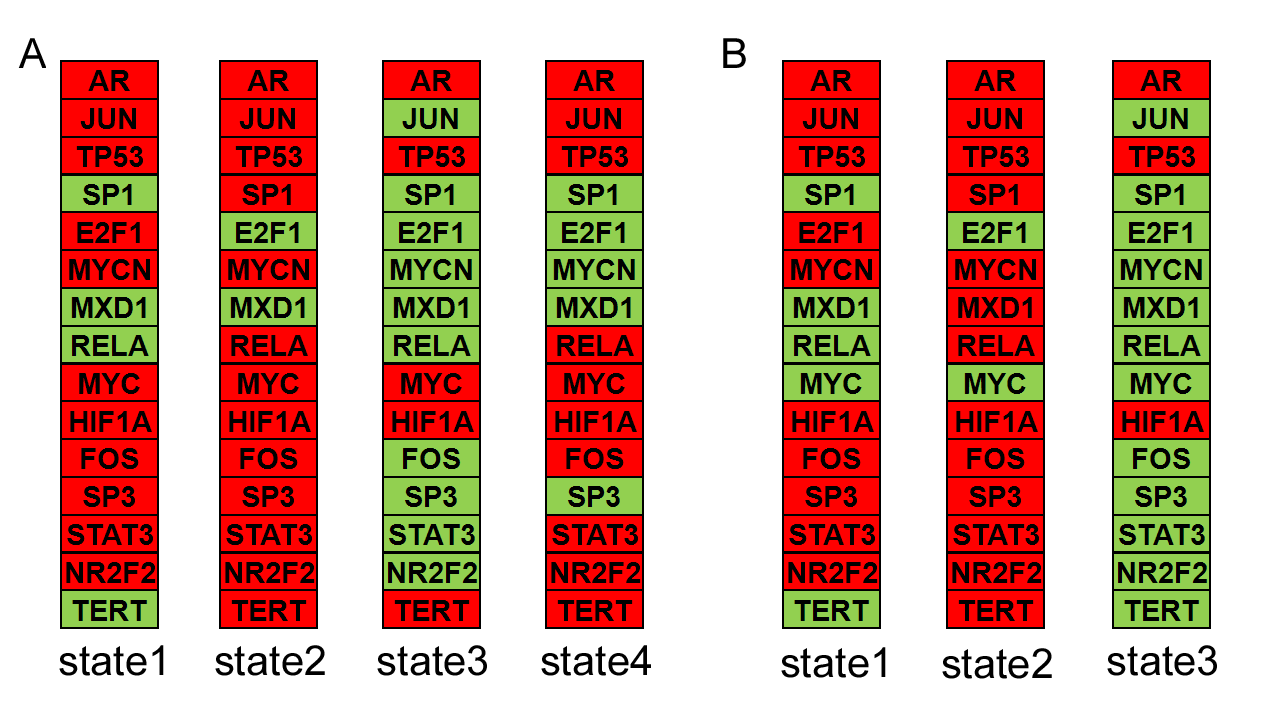


**Figure S6: Attractor states of the model based on the cut-offs FC>2, p<0.01. (A) Basal conditions, (B) *MYC* inhibited.**

The results for the selected model with FC1.5, p<0.01 are detailed in the main text. This model gave the best results overall. However, we also tested a version of the model with FC>1.5-fold, p<0.05. This had 133 interactions. Analysis of the attractor states of this version is shown in figure S7. The basal model had a single attractor state with *TERT*-on as required for acceptance (figure S7A). However, In this case, setting *MYC* to be constitutively off did not result in *TERT* suppression. In contrast, a single attractor was found in which *TERT* remained on (figure S7B). With this set of cut-offs, BIO also increased activity of the *E2F1* promoter with FC=2.1, p=0.013 in addition to the effects on *STAT3* and *FOS* described in the main text. Hence, GSK3 inhibition was modelled by setting *E2F1* constitutively on, *FOS* constitutively off and *STAT3* constitutively on. The resulting limit cycle is shown in figure S7C. There a*r*e no stable *TERT*-on states and the model passes criterion 3 with the additional interesting behaviour of oscillation of *TERT, TP53, NR2F2* and *JUN*. However, although this model passed criteria 1 and 3, it was rejected on criterion 2.


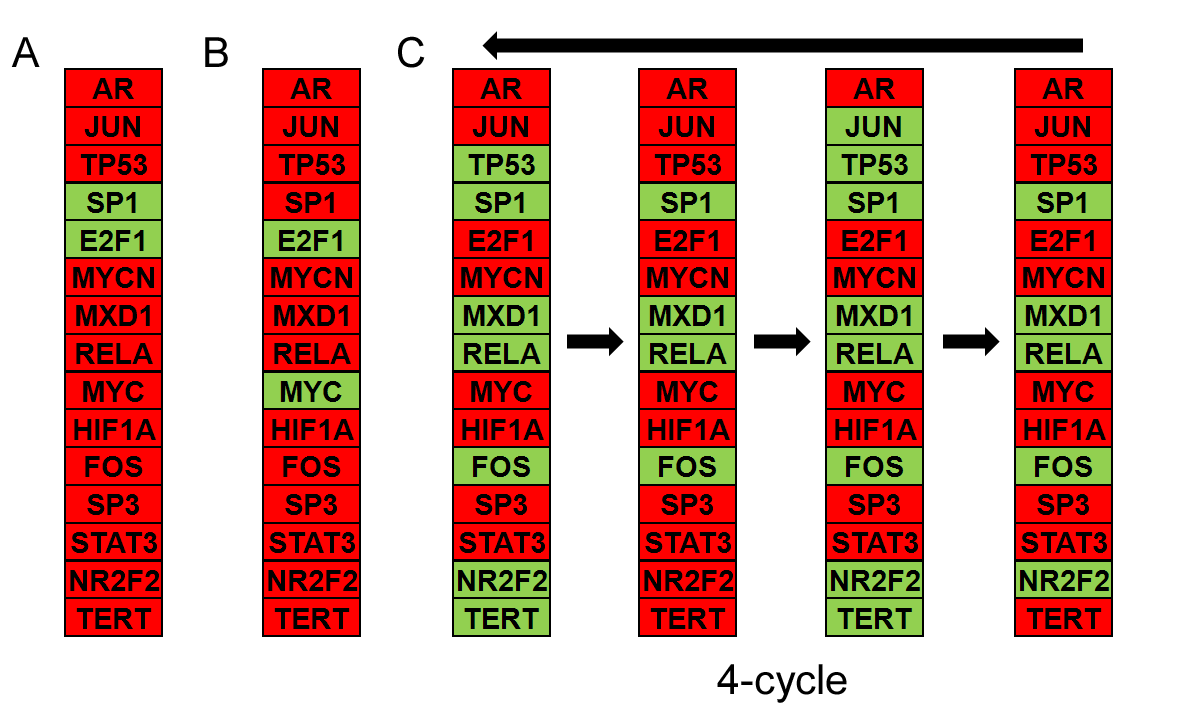


**Figure S7: Attractor states of the model variant FC>1.5, p<0.05. (A) Basal conditions, (B) *MYC* inhibited, (C) BIO simulation.**

We also tested model behaviour when only significance of the transfection screening results is taken into account as the interaction assignment criterion. In comparison with the selected model (FC >1.5, p<0.01), when p<0.01-only is used, a single additional interaction is included in the model. This is a repressive interaction from *HIF1A* to *SP1* (fold change -1.35, p<0.01). In addition to our core selection criteria, we have also tested the behaviour when this interaction is included against the other compounds tested in the main text (figure S8).

Hence, we analysed attractor states of the basal, unpert**u**rbed model (figure S8A), simulation of SU6656 (figure S8B), simulation of FR180204 (figure S8C), simulation of BIO (figure S8D), and simulation of *MYC* suppression (figure S8E). There was no change in the effect of any compound on the promoter panel by eliminating the fold-change cut-off. It can be seen that for each of figures S8A-D, the attractor states of the significance-only model are identical with those described in-text for the original model. Although it is not specifically reported in the text, the 2-cycle with *TERT*-off in both states (figure S8E) is also identical to that obtained when *MYC* is constitutively suppressed in the model incorporating both fold change and significance. Hence, the results obtained for the p<0.01 significance-only model do not differ for any of the key analyses reported.


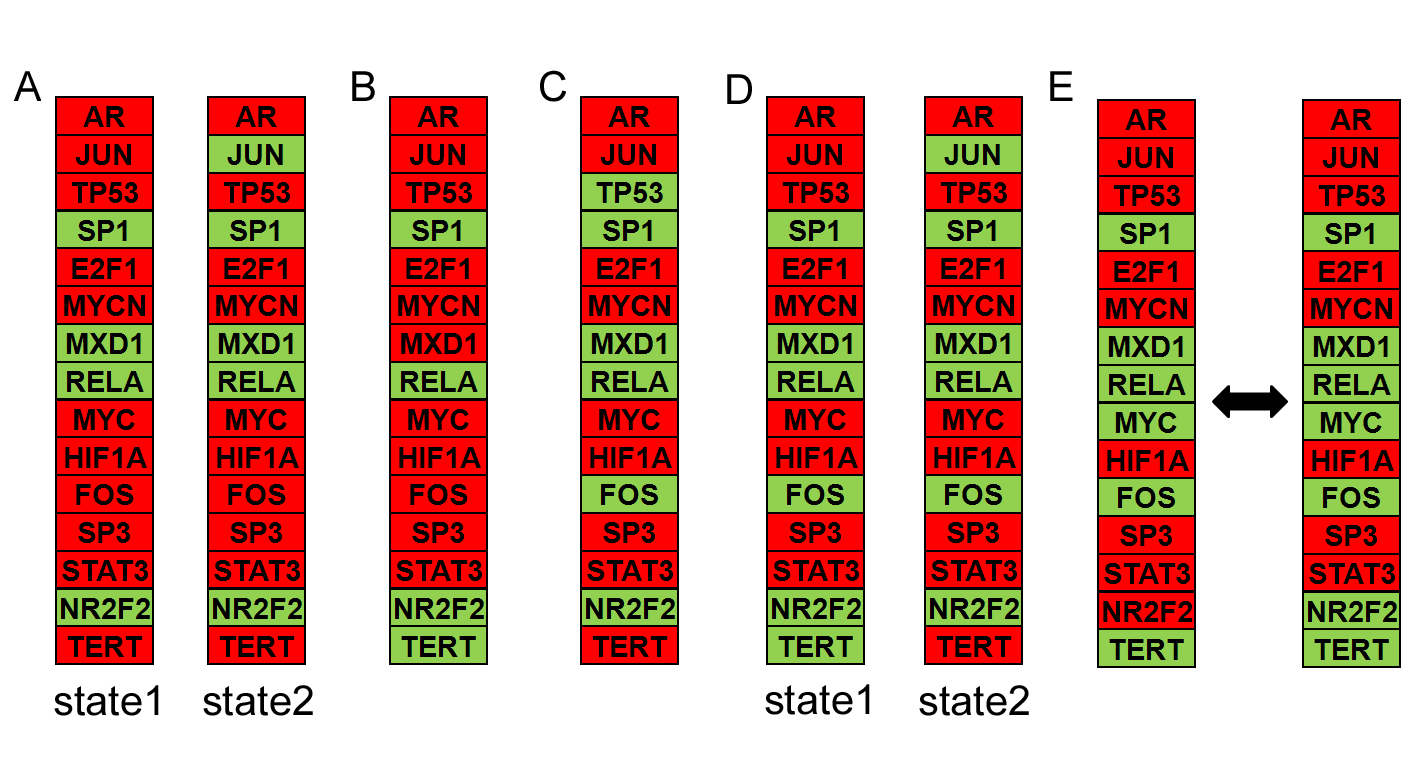


**Figure S8: Attractor states of the significance-only model with p<0.01. (A) Basal states, (B) SU6656 simulation, (C) FR180204 simulation, (D) BIO simulation, (E) *MYC* inhibition.**

We also tested a model variant when p<0.05-only is used. This model had 128 interactions and a complex basal state space comprising 2 limit cycles, neither with *TERT* stable-on (figure S9A). Thus, this model failed on the first selection criterion. Simulation of *MYC* inhibition resulted in a single attractor with *TERT*-off (figure S9B). With p<0.05, the effect of BIO on the promoter panel was activation of *E2F1* and *STAT3* and repression of *FOS*. This rule change produced the 2-cycle in figure S9C in which *TERT* is off in both sub-states. However, since no stable *TERT*-on states were present in the basal statespace, it cannot be concluded that either the *MYC* or BIO rule changes led to *TERT* repression in this model. Hence, the model was rejected.


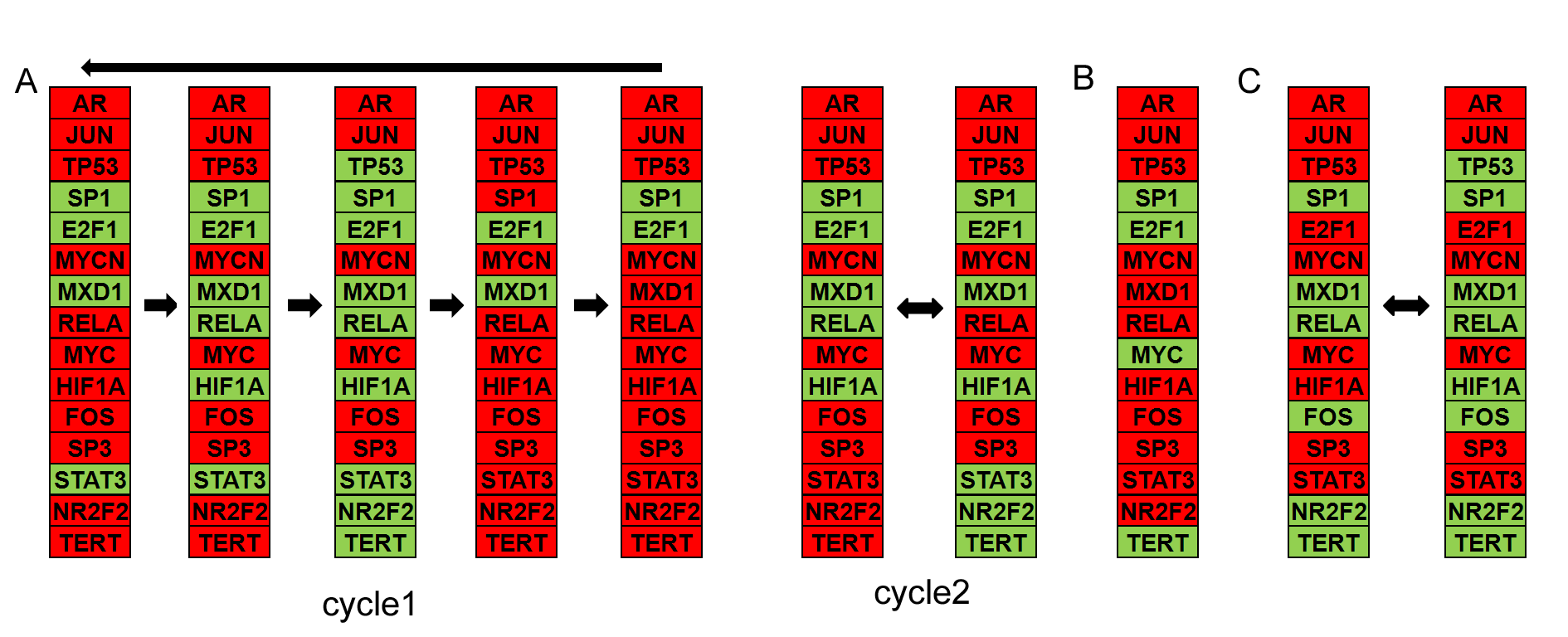


**Figure S9: Attractor states of the significance-only model with p<0.05. (A) Basal states, (B) BIO simulation, (C) *MYC* inhibition.**

# 15-node literature derived model

Although we tested other literature derived models prior to development of the screening approach, in review of the manuscript we also compared the selected model with a new literature based version comprising the same factors. We used MetaCore to define a direct-interactions network for these utilising the network-build pre-filters: interaction-mechanism (effect on expression, transcriptional regulation, excluded all other mechanisms) and interaction-effect (positive, negative, excluded unspecified) (figure S10). This model had 67 interactions. We applied the threshold rule to this network and characterised the resulting Boolean model.


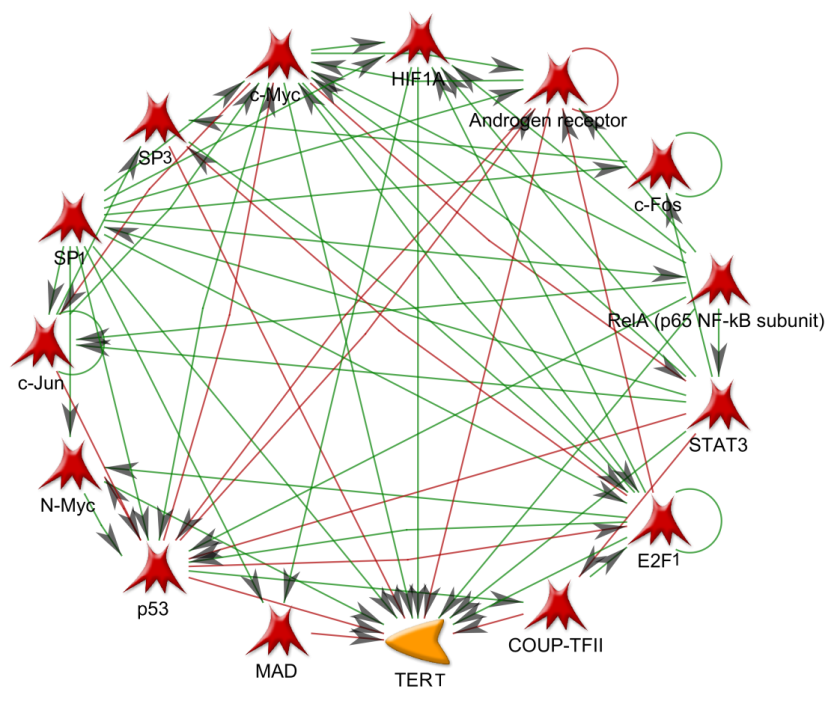


**Figure S10: Literature-derived model comprising the same factors used in the screening-derived model.**

In the basal state, this model has a single attractor in which all nodes are on (figure S11A). Therefore, the model passes criterion 1. Suppression of *MYC* in the model produces a 2-cycle in which the state of *AR* alone is flipping (figure S11B). Again, *TERT* is on; hence, the model fails the second criterion. Simulation of GSK3 inhibition in this model again used the approach based on our transfection screening results for the effect of BIO in the selected model – suppression of *FOS* and activation of *STAT3*. This results in a single steady state in which the only change relative to the basal model is that *FOS* is off (figure S11C). *TERT* is on in this attractor, and the model therefore fails our criterion 3.


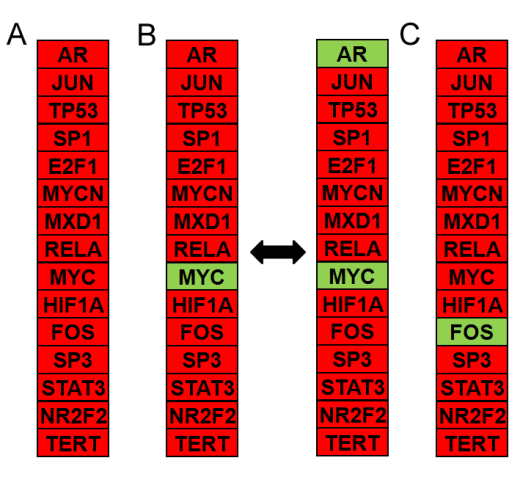


**Figure S11: Attractor states of the literature-derived model comprising the same factors used in the screening-derived model. (A) Basal states, (B) *MYC* inhibition, (C) BIO simulation.**

Finally, in literature searching to verify which interactions from the selected model appear to be novel according to our inclusion/exclusion criteria identified in the paper (tables 2-4), we identified an additional 11 interactions which have previously been reported. We assessed the extent of overlap of the complete set of literature-derived interactions with our own, as described in the text. A total of 47 interactions were shared with 35 being directionally concordant. We also tested a model based only the shared interactions, taking the literature-derived effect (activation or repression) where our results differed. The basal state of this model was a 2-cycle in which TERT flips between the on and off states under control of *MYCN* self-inhibition (figure S12). Since *TERT* was unstable, this was not further investigated.


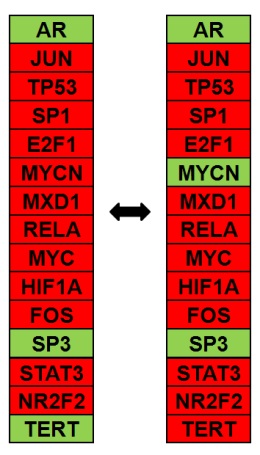


**Figure S12: Basal state of the literature/data overlap model.**

# The role of *STAT3*

As noted in the text, we retained *STAT3* as a *TERT* activator, though it did not reach the significance threshold (p=0.078, FC=1.57). We have previously detected *STAT3* binding to the *TERT* promoter and other studies have indicated that *STAT3* does increase *TERT* expression. In preparation of this study we performed ChIP analysis of *STAT3* again. We analysed its binding to the *TERT* promoter and to the promoters of *RELA* and *SP1* whose promoters it did stimulate significantly in our transfection screen (*RELA*, FC=2.05, p=0.006; *SP1*, FC=2.1, p=0.023). These results are given in figure S13. As in our previous studies, *STAT3* was again significantly enriched at the *TERT* promoter. We also detected significant binding at those of *RELA* and *SP1* as expected.


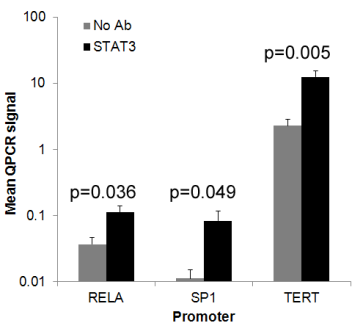


**Figure S13: Chromatin IP analysis of *STAT3* binding to the *TERT*, RELA, and *SP1* promoters. Mean ± SEM of three biological repeats. QPCR analysis of each repeat was performed using technical triplicates.**

Hence, STAT3 protein does appear to bind the endogenous *TERT* promoter in these cells. Notably, our ChiP primers detect STAT3 protein enrichment even though they are focused on a region of the core promoter which, as in our construct lacks direct STAT3 binding sequences. It may be that expressed STAT3 interacts with this region through a protein complex rather than by direct binding and a strong stimulatory effect may require the presence of other unknown factors.

Because of the nature of the threshold rule, which is a balance between activating and repressive interactions, in model development we felt that a network having an evenly balanced number of *TERT* activators and repressors would likely give better results. In preference to reducing the model size to achieve that balance, we therefore retained *STAT3* as an activator throughout model selection. We have, however, tested the performance of a model based on the selected FC>1.5, p<0.01 variant in which *STAT3* is removed from the network completely. We have tested the attractor states of this 14-node model against each of our key results from the main text obtained using the full 15-node network (figure S14).


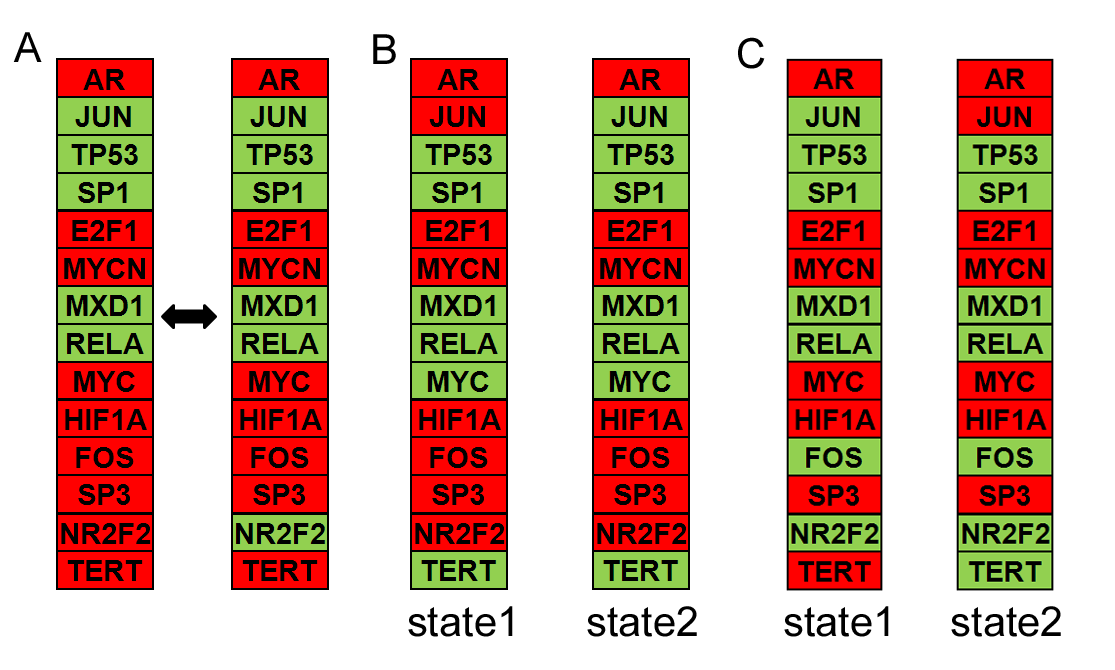


**Figure S14: Attractor states of the FC>1.5, p<0.01 model with *STAT3* removed. (A) Basal states, (B) *MYC*-inhibited, (C) BIO simulation.**

The basal state of this model is a 2-cycle with *TERT*-on in both sub-states which satisfies our first criterion for model selection of *TERT*-on stability (figure S14A). Simulation of *MYC* inhibition produced 2 attractors, both with *TERT*-off, which satisfies our second criterion (figure S14B). Simulation of the effect of BIO (which in this case is suppression of *FOS* only, since *STAT3* is removed) produced 2 attractors (figure S14C), one with *TERT*-on (state 1) and the other with *TERT*-off (state 2). Hence, this model also satisfies selection criterion 3. This result is similar to the effect obtained with the complete model. Notably, the states of all entities are the same under these rules in both models except for *TP53*. However, in the model without *STAT3*, the largest state (state 1) has *TERT*-on, rather than off, likely as a result of loss of *TP53* activation. Thus, this model predicts that BIO will cause some *TERT* repression but noise will tend to select for *TERT* expression, though this still is dependent on the state of *JUN* as in the complete model and in line with our previously reported results (Bilsland et al., [PLoS One.](http://www.ncbi.nlm.nih.gov/pubmed/19649288) 2009 Jul 31; 4(7):e6459).

Under simulation with FR180204, 2 attractors are obtained: one with *TERT*-off, one with *TERT*-on (figure S15A). The off-state is associated with a slightly larger proportion of statespace (56.6%). Thus, the model predicts that the ERK inhibitor should result in at least some *TERT* repression, which is not in line with our QPCR results after treatments with this compound (main text, figure 3). However, under simulation of SU6656, a single state with *TERT*-off is obtained (figure S15B), as in the complete model and consistent with our QPCR results reported in the text (main text, figure 3).


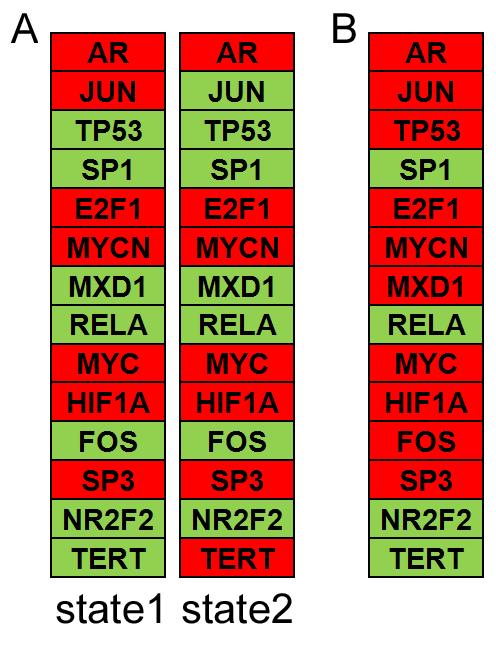


**Figure S15: Attractor states of the FC>1.5, p<0.01 model with *STAT3* removed. (A) FR180204 simulation, (B) SU6656 simulation.**

We also tested how well the model fits the data on *MYC*-dependent *TERT*-repression robustness as compared with the complete model. We performed the analysis of sequential constitutive activation or repression of each node alone, or in the background of constitutive *MYC* suppression as in figure 5C of the main text. These results are given in figure S16. In this analysis using the complete model, we obtained the prediction that *MYC*-dependent *TERT*-repression is largely reversible when *AR* is simultaneously knocked-down and is also partially reversible by *SP3* siRNA. We confirmed these predictions by RNAi/QPCR in figure 5D-E of the main text.

In the model without *STAT3*, we find the major difference in this analysis is one of magnitude. Both nodes still play a role in repression reversal (emergence of grey bars), but *SP3* has the greater effect. Hence, the model with *STAT3* conforms better to our data but the general predictions are retained. The effects of the single constitutive mutations *TP53*-on, *MYCN*-off, *MXD1*-on, *HIF1A*-off and *FOS*-off were also all detected in both models, though again at different magnitudes (compare figure 5C in the text). All other results were similar or identical. Hence, the main divergent results with this model are the effect of the ERK inhibitor and the magnitude of reversal by *SP3* or *AR*. The selected model does perform better, but most essential results are still captured in the *STAT3*-deleted model.


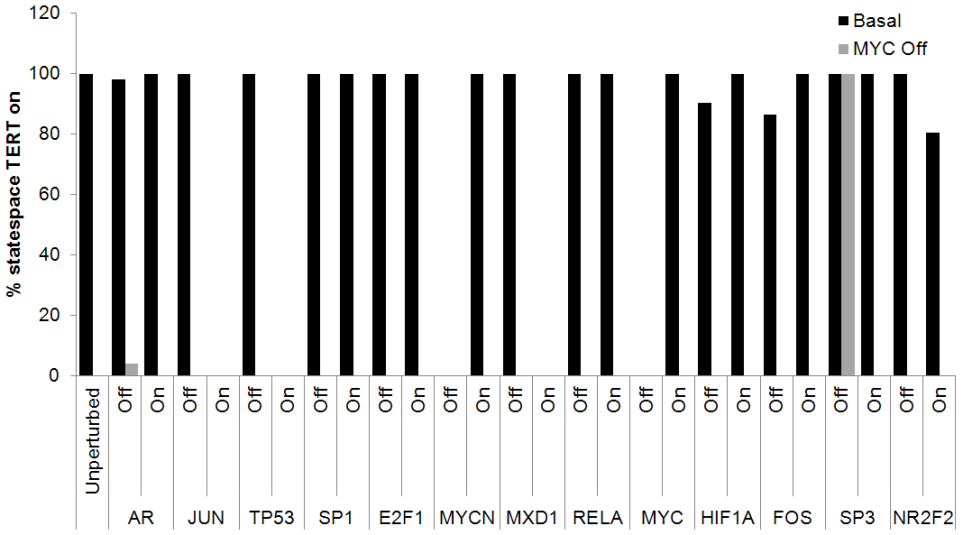


**Figure S16: Analysis of *TERT* expression robustness in the STAT3-negative model. Nodes were set to be constitutively on or off, either alone or in combination with constitutive *MYC*-off. The fraction of statespace associated with TERT-on attractors was determined for each case.**

# Extracting AM/RM

For network size N, let A be the N x N adjacency matrix which is equal to the signed weight matrix, A = W = {a: a_ij_ = ω_ij_}. Let v be the index of the vertex whose activation or repression subnetwork it is desired to extract. Furthermore, let P be a 1 x N partition on the network which indexes repressors (1), activators (2), or v (3). We use construction matrix G, initially equal in dimension to A. G is initialised with all elements zero and then populated with the required edge sets. We consider the case where v is a sink for all other vertices. In both cases, loops are excluded. First perform:

loopsAndPartitions (A, P, N, v) {

FOR i equal 1 to N

SET A[i][i] to 0

CASE A[i][v] OF

-1: SET P[i] equal 1

1: SET P[i] equal 2

ENDCASE

ENDFOR

SET P[v] equal 3

RETURN (A, P)

}

We now can extract the activation module. We call the functions sumRows and sumColumns which, respectively, return the 1 x N vectors rows and cols in which the element rows[i] holds the absolute tally of non-zero elements in the i^th^ row of G. The element cols[j] holds the absolute tally of non-zero elements in the j^th^ column of G. We also call the function min, which returns the value of the smallest element of the 1 x N vector nodeDegrees. Finally, we call deleteRow and deleteCol. These functions shrink the dimensions of G. Note that N is also decreased according to the dimension of G.

ActMod (A, P, N) {

INIT G

FOR i equal 1 to N

FOR j equal 1 to N

CASE P AND A OF

P[i] equal 2 AND P[j] equal 2 AND A[i][j] equal 1: SET G[i][j] equal 1

P[i] equal 2 AND P[j] equal 1 AND A[i][j] equal -1: SET G[i][j] equal -1

P[i] equal 2 AND P[j] equal 3: SET G[i][j] equal 1

P[i] equal 1 AND P[j] equal 3: SET G[i][j] equal -1

ENDCASE

ENDFOR

ENDFOR

REPEAT

SET deletions equal 0

COMPUTE rows[] as CALL sumRows(G)

COMPUTE cols[] as CALL sumColumns(G)

COMPUTE nodeDegrees[] as vector sum of rows and cols

SET minDegree equal CALL min(nodeDegrees[])

FOR i equal N to 1

IF nodeDegrees[i] < 2

CALL deleteRow(G[i][])

CALL deleteColumn(G[][i])

INCREMENT deletions

ENDFOR

SET N equal COMPUTE N minus deletions

UNTIL minDegree > 1

RETURN (G)

}

To implement RepMod, it is sufficient to redefine the first two edge sets specified by CASE:

P[i] equal 1 AND P[j] equal 1 AND A[i][j] equal 1: SET G[i][j] equal 1

P[i] equal 1 AND P[j] equal 2 AND A[i][j] equal -1: SET G[i][j] equal -1
